# An umbrella review of adverse effects associated with antipsychotic medications: the need for complementary study designs

**DOI:** 10.1016/j.neubiorev.2023.105454

**Published:** 2023-12

**Authors:** Rachel T.S. Chow, Daniel Whiting, Louis Favril, Edoardo Ostinelli, Andrea Cipriani, Seena Fazel

**Affiliations:** a Department of Psychiatry, University of Oxford, Oxford, England, UK; b Institute of Mental Health, University of Nottingham, Nottingham, England, UK; c Nottinghamshire Healthcare NHS Foundation Trust, Nottingham, England, UK; d Faculty of Law and Criminology, Ghent University, Ghent, Belgium; e Oxford Health NHS Foundation Trust, Warneford Hospital, Oxford, England, UK

**Keywords:** Adverse effects, Antipsychotics, Psychosis, Psychopharmacology, Sedation, Metabolic

## Abstract

Antipsychotic medications are widely prescribed in psychotic illnesses and other mental disorders. Effectiveness is well-established, particularly for reducing symptoms such as delusions and hallucinations, but can be impacted by tolerability. Adverse effects are wide-ranging, and vary between antipsychotics, which is clinically important. This umbrella review aimed to comprehensively summarise the extent and quality of evidence for adverse effects associated with antipsychotic use in people with mental disorders. We included 32 meta-analyses of randomised trials and observational studies. The overall robustness of reported associations was considered in terms of review quality, heterogeneity, excess significance bias, and prediction intervals. Using this approach, endocrine and metabolic, movement-related, and sedation and sleep problems were the clinical domains with strongest evidence. The overall quality of included meta-analyses was low, and individual adverse effects were not typically examined in meta-analyses of both randomised trials and observational study designs. Future reviews should focus on adhering to methodological guidelines, consider the complementary strengths of different study designs, and integrate clinically relevant information on absolute rates and severity of adverse effects.

## Highlights

• We reviewed 32 meta-analyses of adverse effects associated with antipsychotic use.
• Endocrine, metabolic, movement and sedation effects had the most consistent evidence.
• Overall the quality of included meta-analyses was low.
• Future studies should compare data from different study designs.

## 1 Introduction

Antipsychotics remain the drug class with the most robust evidence of effectiveness in psychotic disorders such as schizophrenia (Ceraso et al., 2022). They are also widely used in a range of other mental disorders, including in severe depression and bipolar disorder, and as off-licence treatments for symptom management in personality disorder, dementia, and some anxiety disorders. Altogether, nearly 800,000 individuals are prescribed these medications in England, (NHS Business Services Authority, 2020) and prevalence is estimated at 1.7% in the US, (Dennis et al., 2020) with increasing rates in children and adolescents (Radojčić et al., 2023).

In psychotic illness, first generation antipsychotics (also known as typical or conventional) exert their therapeutic effects most prominently in the reduction of positive symptoms such as delusions or hallucinations, through postsynaptic blockade of dopamine receptors. Second generation (atypical) antipsychotics are characterised by more weighting towards serotonergic modulation (Li et al., 2016). These profiles account for different adverse effect profiles between the two groups.

The clinical effectiveness of antipsychotics in symptom reduction is complicated by a range of potential adverse effects. The traditional division is that for first generation antipsychotics, dopamine receptor occupancy in nigrostriatal pathways is linked to musculo-skeletal problems, whereas the multi-receptor profile of second generation antipsychotics is associated with more metabolic adverse effects. A range of other potential effects, from cardiac effects to hyperprolactinaemia, may differ across individual antipsychotics. Factors such as anti-cholinergic and anti-histaminergic action have also been demonstrated to influence the adverse effect profiles of antipsychotics (Kaar et al., 2020). However, in contrast to the investigation of efficacy profiles, adverse effects of antipsychotics are not reported consistently enough to allow for a robust classification under the ‘Dose, Time and Susceptibility’ framework (Aronson and Ferner, 2003). As some adverse effects have serious health consequences, and these medications are widely prescribed, this needs clarification.

An effective way to address such a broad evidence gap is with an umbrella review, which comprehensively summarises and assesses the quality of systematic reviews (Fusar-Poli and Radua, 2018). This can provide new insights and a clearer overview of fields with a large or conflicting literature. Two relevant umbrella reviews on the adverse effects associated with antipsychotic use in adults have previously been undertaken. One reviewed the physical health effects of antipsychotic, antidepressant and mood stabilising medication (Correll et al., 2015). However, this was undertaken when umbrella review methodology was in its infancy, used unclear inclusion criteria, and did not include a quality assessment of included reviews. A second umbrella review of adverse effects of antipsychotics (Papola et al., 2019) included six systematic reviews of 58 observational studies, but its scope was limited to observational studies (not randomised controlled trials [RCTs]) of six life-threatening adverse events. Other umbrella reviews have considered pharmacological interventions more widely in mood disoders (Croatto et al., 2023) and child and adolescent populations, (Solmi et al., 2020) and focussed on metabolic adverse effects in children and adolescents treated with antipsychotics (Carnovale et al., 2023).

The current umbrella review sought to address evidence gaps and limitations in previous work by considering a broader range of all reported adverse effects associated with antipsychotic medication, and by including systematic reviews and meta-analyses of both randomised and non-randomised experimental study designs. The aims were to 1) map and summarise the extent and quality of evidence for adverse effects associated with antipsychotic use, 2) compare risk across adverse effect categories, and 3) identify the key gaps in existing literature for future work to target.

## 2 Methods

This umbrella review followed PRISMA harms reporting guidelines (Zorzela et al., 2016) and the PRIOR statement for reviews of healthcare interventions (Appendix 1) (Gates et al., 2022). The protocol was registered on PROSPERO (CRD42020223706).

### 2.1 Data sources

A systematic search was conducted of PubMed, Embase, PsycINFO, Scopus, CINAHL and the Cochrane Library of Systematic Reviews, from inception to 30th July 2023. The search strategy incorporated the names of individual antipsychotic medications and search strings comprising of database-specific indexing terms (e.g. MeSH terms) attached to adverse effect-related subheadings. Study design filters developed by the National Library of Medicine (National Library of Medicine, 2019) and British Medical Journal (BMJ Best Practice, n.d.) were incorporated in the search strategy to identify relevant studies on PubMed and Embase respectively. See Appendix 2 for the full search strategy for all databases.

### 2.2 Record screening and eligibility

We aimed to include systematic reviews with meta-analyses of RCTs or observational studies with a cohort, case-control, nested case-control or cross-sectional design, which 1) reported associations between adverse effects and any of the 32 antipsychotic medications of interest (see below), 2) examined antipsychotic prescription as either monotherapy or combination therapy at any dose, 3) investigated specified adverse effects associated with antipsychotic use in human populations of any age with any psychiatric illness or medical condition, 4) reported adverse effects with study-level data that allowed for the calculation of risks using odds ratios (ORs) or relative risks (RRs), and 5) measured adverse effects as primary or secondary outcome.

Antipsychotic medications of interest were determined based on the British National Formulary, United States Pharmacopeia-National Formulary (USP 43–NF 38), and ‘Medicines used in psychotic disorders’ from the WHO Model List of Essential Medicines (2019) (World Health Organization, 2019). These were: acepromazine, amisulpride, aripiprazole, asenapine, benperidol, cariprazine, chlorpromazine, clozapine, droperidol, flupentixol, fluphenazine, haloperidol, levomepromazine, loxapine, lurasidone, molindone, olanzapine, paliperidone, pericyazine, perphenazine, pimozide, prochlorperazine, promazine, quetiapine, risperidone, sulpiride, thioridazine, thiothixene, trifluoperazine, triflupromazine, ziprasidone, and zuclopenthixol. Reviews examining in-utero exposure or primarily investigating antipsychotic overdose were not included.

Several criteria were used to focus the range of reported adverse effects. First, adverse effects with data obtained from fewer than 3 primary studies were excluded due to the limitations of estimating effect size heterogeneity in these cases (von Hippel, 2015). Second, where appropriate, clinical diagnoses (e.g. extrapyramidal disorder) and their synonymous or associated symptoms (e.g. akathisia, dystonia) were considered as the same outcome. Third, to ensure clinical relevance of the adverse effects considered, observations that were not anchored in diagnoses, or were not of a clinically relevant threshold or defined in a reproducible manner (e.g. increased or decreased appetite, increased or decreased duration of sleep, without further specification) were excluded. We limited the range of cause-specific mortality outcomes to suicidal mortality, cardiac death and sudden death.

We aimed to examine antipsychotic use in predominantly community settings, as these likely represent more stable prescription patterns, whereas treatment in the acute phase may more likely be complicated by polypharmacy or underreporting of adverse effects due to symptom severity. Therefore, meta-analyses which specified they included only inpatient samples, and/or reported non-stratified data that included > 30% primary studies with institutionalised samples were excluded (threshold set a priori through discussion within research team to balance the number of studies included with representativeness of samples). Meta-analyses on surgical or palliative care patients were also excluded as these are distinct populations.

Where multiple meta-analyses of effect sizes for the same antipsychotic medication / adverse effect / study type / population combination were identified, only one of the meta-analyses was included. See Appendix 3 for the process for this selection. This eliminated the potential for there to be any overlap between primary studies (i.e. double-counting) incorporated in pooled effect-sizes.

### 2.3 Data extraction

Full texts of potentially relevant records were retrieved and screened using a standard template. For each included systematic review and meta-analysis, data extracted included: design of primary studies; number of databases searched; year range of review; number of primary studies; study location; settings; number of participants; participant age; participant diagnoses; type(s) of antipsychotics; adverse effects; effect sizes with 95% confidence intervals; and for reviews of observational studies, the number of cases exposed to the adverse outcome and the number of cases without exposure to the adverse outcome. RC undertook data extraction, which was independently duplicated for a random 20% of included studies by LF and any discrepancies resolved by a third reviewer (DW).

### 2.4 Analysis

To account for high heterogeneity, wherever possible, summary effect estimates and corresponding 95% confidence intervals from included meta-analyses were re-estimated using the Dersimonian-Laird random-effects model. For 9 included meta-analyses (28%), insufficient primary study-level data was reported to allow this re-estimation, and effect sizes were reported directly. For each adverse event, associations with zero events in the exposed and non-exposed groups were excluded during re-estimation. If a meta-analysis reported multiple associations respectively for different types of antipsychotics, doses and/or patient characteristics, these associations were pooled to calculate an overall summary effect estimate and heterogeneity statistics using the Dersimonian-Laird random-effects model. The respective effect sizes and information on individual antipsychotics, dose-response relationships, duration, and patient characteristics were discussed and investigated via subgroup analyses.

For observational studies, where adverse effect incidence is typically lower, ORs and RRs are approximately equivalent. For RCTs, incidence is typically higher, and so where meta-analyses reported effect sizes other than RRs, if sufficient information was reported this was used to re-estimate effect size as RRs. Conversion between ORs and RRs requires baseline prevalence data, and as this was not typically available, universal conversion was not undertaken.

### 2.5 Quality and credibility assessment

The methodological quality of included reviews was critically appraised using the AMSTAR 2 tool. Of the 16 items, 7 domains are regarded as critical (items 2, 4, 7, 9, 11, 13 and 15) (Shea et al., 2017). Each item was rated “yes, ”partial yes” or “no”. To summarise quality, an inadequate rating (i.e. scoring “no”) on a critical domain was defined as a critical flaw, and on a non-critical domain defined as a non-critical weakness. Based on this, overall confidence in meta-analyses’ findings were rated as high (no or one non-critical weakness), moderate (more than one non-critical weakness), low (one critical flaw with or without non-critical weakness) or critically low (more than one critical flaw with or without non-critical weaknesses) (Shea et al., 2017). Quality assessment was undertaken by RC, and checked independently for a random 20% of studies independently by LF, with any discrepancies resolved by a third reviewer (DW).

Two adaptations of the standard AMSTAR 2 criteria were made: 1) for item 7, the inclusion of a full list of references was not practical in most cases, (Hailes et al., 2019) and so this criterion was modified to state that a summary of excluded studies, in the form of PRISMA flowchart or equivalent description in the results section, would suffice; 2) for item 15, investigation of publication bias via graphical or statistical tests was not possible or appropriate for some reviews, and so this criterion was modified to state that specific attempts to identify publication bias would suffice.

### 2.6 Overall evidence consistency and robustness

We used an overall score for consistency and robustness developed for umbrella reviews (Hailes et al., 2019). Each identified adverse outcome was assigned a score on four criteria: between-study heterogeneity (< 50% = 1, 50–75% = 0.5, > 75% = 0), prediction intervals (rejects the null hypothesis = 1, no = 0), excess significance (no = 1, yes = 0), and AMSTAR 2 rating (high = 1, moderate = 0.5, low = 0, critically low = 0). See Appendix 5 for full statistical methods. The four quality scores were then summed to determine an aggregate overall rating within the range of 0–4. Composite scores of 3 or 4 (out of 4) indicate high overall consistency/robustness of evidence for the respective adverse outcome, with scores less than 1.5 indicating low consistency/robustness.

### 2.7 Deviations from protocol

Deviations from the pre-registered protocol are detailed in Appendix 4.

## 3 Results

### 3.1 Characteristics of included studies

In total, of 6206 unique records identified, 895 full texts were scrutinized, and 32 meta-analyses were included (Appendix 6). The included reviews were published between 2007 and 2023 and reported on 38,661,668 participants and 1438 associations from primary studies. Eligible systematic reviews provided effect size data for 270 adverse effects (see Appendix 7 for all excluded adverse effects with reasons and Appendix 8 for eligible but excluded studies). Of the 166 associations for which data extraction was double checked, there were only 2 disagreements in effect sizes or confidence intervals, which were clarified. The weighted kappa of quality rating duplication was 0.89.

Twenty-one (66%) of the included articles were meta-analyses of RCTs, (Bai et al., 2020, Côrte-Real et al., 2023, Demyttenaere et al., 2019, Derry and Moore, 2007, Hay et al., 2015, Kishimoto et al., 2022, Lao et al., 2016, Lin et al., 2023, Luan et al., 2017, Ma et al., 2014, Maglione et al., 2011, Maher et al., 2011, Nussbaum and Stroup, 2008, Nussbaum and Stroup, 2012, Ostuzzi et al., 2021, Polcwiartek et al., 2015, Reichelt et al., 2023, Romeo et al., 2018, Rotella et al., 2020, Schneider-Thoma et al., 2018, Trinchieri et al., 2021) reporting data on 38 adverse effects and over 1176 unique associations, with sample sizes ranging from 517 to 79,544 (total N = 311,319 participants). One RCT meta-analysis was on antidepressant augmentation of antipsychotic medication in treatment-resistant depression, while the rest primarily examined antipsychotic monotherapy. Reviews considered different diagnostic groups, including schizophrenia-spectrum disorders (9 meta-analyses), bipolar disorders (k = 10), major depressive disorder (k = 7), dementia (k = 4), and off-label use in personality disorder and other mental disorders (k = 2). Table 1 presents the characteristics of included RCT meta-analyses, including comparators.

**Table 1:** Characteristics of included RCT meta-analyses.

Author	Year	Population age	Diagnoses or population characteristics	Antipsychotic (s): Dose / Comparator	Follow-up duration	Adverse effect	*k*	*N*
Bai et al.	2020	≥ 18 years	Bipolar disorder	Aripiprazole: 5–30 mg/d or 400 mg OM Asenapine: 10–20 mg/d Cariprazine: 0.25–12 mg/d Haloperidol: 5–30 mg/d Lurasidone: 20–120 mg/d Olanzapine: 5–20 mg/d Paliperidone: 3–12 mg/d Quetiapine: 150–800 mg/d Risperidone: 1–6 mg/d or 12.5–50 mg/2 weeks Ziprasidone: 40–160 mg/d / Placebo	3–104 weeks	Clinically significant weight gain (≥7%)	49	17,167
						Somnolence	47	16,269
Côrte-Real et al.	2023	≥ 10 years	Major depressive disorder or bipolar disorder	Aripiprazole: 2–30 mg/d Cariprazine: 0.25–3 mg/d Lurasidone: 20–1120 mg/d Olanzapine: 2.5–20 mg/d Quetiapine: 150–600 mg/d Risperidone: 25–50 mg/d / Placebo	6–52 weeks	Treatment-emergent mania	21	8075
Demyttenaere et al.	2019	≥ 18 years	Schizophrenia, schizoaffective disorder, bipolar disorder or major depressive disorder	Asenapine: 2.5–10 mg BID / Placebo Lurasidone monotherapy or combined with lithium or valproate: 20–120 mg Cariprazine monotherapy or combined with antidepressants: 0.25–9 mg / Placebo or placebo plus lithium, valproate or antidepressants	3–46 weeks	Extrapyramidal disorder	33	12,864
Derry & Moore	2007	Depressive episodes: 36–42 years Mania or mixed episodes: 35–43 years	Bipolar I or II disorder with current depressive, manic or mixed episodes	Olanzapine monotherapy or combined with mood stabilisers: 5–20 mg/d / Placebo or mood stabilisers plus placebo Quetiapine: 100–800 mg/d Risperidone combined with mood stabilisers: 1–6 mg/d / Placebo or placebo plus mood stabilisers	≥ 3 weeks	Depression	6	1266
Hay et al.	2015	≥ 18 years	Schizophrenia or schizoaffective disorder	Asenapine: 5–10 mg BID / Placebo	6 weeks	Clinically significant fasting glucose level	3	641
Kishimoto et al.	2022	Mean age (AP monotherapy): 45.2 ± 7.6 years Mean age (AP adjunctive therapy): 45.4 ± 5.2 years	Major depressive disorder	Amisulpride: 50 mg/d Quetiapine: 140–177^a^ or fixed doses of 50, 150 or 300 mg/d / Placebo Quetiapine combined with antidepressants: 150–310^a^ or fixed doses of 50, 150, or 300 mg/d Ziprasidone combined with antidepressants: 98^a^ or fixed doses of 80 or 160 mg/d / Placebo plus antidepressants	1–52 weeks	Anxiety	7	1164 patients
				Amisulpride: 50 mg/d Quetiapine: 140–177^a^ or fixed doses of 50, 150 or 300 mg/d Ziprasidone: 81 or 114 mg/d^a^ / Placebo Aripiprazole combined with antidepressants: 2.5–12^a^, 7–10^b^, or fixed doses of 2, 3, or 5 Quetiapine combined with antidepressants: 150–310^a^ or fixed doses of 50, 150 or 300 mg/d / Placebo plus antidepressants		Blurred vision	6	1540 patients
Lao et al.	2016	≥ 18 years (Mean range: 35.5–46.4)	Schizophrenia, bipolar I mania and depression or major depressive disorder	Cariprazine monotherapy or combined with antidepressants: 0.75–12 mg/d / Placebo or placebo plus antidepressants	3–8 weeks	Hypotension	7	3207
						Suicidal ideation	6	2900
Lin et al.	2023	≥ 18 years	Individuals with generally good health or diseases who had their sleep quality outcomes assessed	Quetiapine: 50–300 mg / Placebo	6–52 weeks	Sexual dysfunction	10	4146
Luan et al.	2017	Adults	Treatment-resistant depression	Olanzapine monotherapy or combined with fluoxetine: 6–18 mg/d / Placebo or placebo plus fluoxetine	8–12 weeks	Hypersomnia	2	1231
						Peripheral oedema	4	1978
Ma et al.	2014	40–97 years	Dementia	Aripiprazole: 2–10 mg/d Olanzapine: up to 17.5 mg/d Quetiapine: up to 600 mg/d Risperidone: up to 200 mg/d / Placebo	6–12 weeks	Gait abnormality	10	2845
				Aripiprazole: 2–15 mg/d Olanzapine: 2.5–15 mg/d Quetiapine: 25–600 mg/d Risperidone: 0.5–2 mg/d / Placebo		Injury	10	3370
Maglione et al.	2011	18–64 years	Off-label antipsychotic use in adults with anxiety, eating disorders, depression, OCD, PTSD, personality disorders, and/ or substance abuse	Olanzapine: * Quetiapine: * Risperidone: * Ziprasidone: * / Placebo	*	Endocrine abnormalities (except diabetes)	5	911
Maher et al.	2011	18–64 years	Off-label antipsychotic use for any diagnosis (adults: anxiety, eating disorders, depression, OCD, PTSD, personality disorders, and/ or substance abuse; older adults: dementia)	Olanzapine: * Quetiapine: * Risperidone: * / Placebo	*	Diabetes mellitus	8	4046
		≥ 18 years		Aripiprazole: * Olanzapine: * Quetiapine: * Risperidone: * Ziprasidone: * / Placebo		Fatigue	30	8613
						Sedation	71	15,760
		≥ 65 years	Off-label antipsychotic use in older adults with dementia	Aripiprazole: 2–15 mg/d Olanzapine: 1–15 mg/d Quetiapine: 25–600 mg/d Risperidone: 0.5–2.5 mg/d / Placebo	6–12 weeks	Stroke	11	3381
Nussbaum & Stroup	2008	≥ 18 years	Schizophrenia	Paliperidone: 3–15 mg/d / Placebo	2 or 6 weeks	Hyperkinesia	4	1360
				Paliperidone: 6–12 mg/d / Placebo	2 or 6 weeks	Hypertonia	5	1225
Nussbaum & Stroup	2012	≥ 18 years	Schizophrenia	Paliperidone: 37–234 mg/4 weeks / Placebo	13–33 weeks	Anxiety	5	2180
						Hypertension	5	2180
						Menstrual disorder	5	762
						Musculoskeletal pain	5	2180
						Rash	5	2180
						Suicide attempt	5	2178
				Paliperidone: 39–156 mg/4 weeks	13 weeks	Pneumonia	1	517
Ostuzzi et al.	2021	Mean: 35.0–57.1 years	Nonaffective psychotic disorder	Aripiprazole: 10–30 mg/d or 300–400 mg/4 weeks Fluphenazine: 50 mg/d^a^ Olanzapine: 150 or 300 mg/2w or 405 mg/4 weeks Paliperidone: 78–234 mg/4 weeks or 175–525 mg/2 weeks / Placebo	12–65 weeks	Hyperprolactinaemia	8	2793
Polcwiartek et al.	2015	6–95 years	Schizophrenia, schizoaffective disorder, bipolar disorder, Tourette syndrome, autism, Alzheimer’s disease	Aripiprazole: 2–30 mg/d / Placebo	1 day–10 weeks	Electrocardiogram abnormalities	18	6106
Reichelt et al.	2023	Mean: 13–83 years	Schizophrenia, bipolar disorder, dementia, major depression, PTSD, and borderline personality disorder	Aripiprazole: 2–30 mg/d^a^, 52.5 or 77.5 mg/week, or 300 or 400 mg/4 weeks Asenapine: 5–20 mg/d^a^ Cariprazine: 8.8 mg/d^a^ Haloperidol: 5–16 mg/d^a^ Lurasidone: 20–120 mg/d^a^ Olanzapine: 1–40515 mg/d^a^, 210 or 300 mg/2 weeks, or 405 mg/4 weeks Paliperidone: 6–337 mg/d^a^ Quetiapine: 50–800 mg/d^a^ Risperidone:0.95–27mg/d^a^ or 25–75 mg/2 weeks Ziprasidone: 160 mg/d^a^	0.14–104 weeks	Seizures	44	14,705
Romeo et al.	2018	All age groups	Major depressive disorder or bipolar depression with depressive or mixed mania episodes	Aripiprazole: > 5 mg/d Cariprazine: > 2.5 mg/d / Placebo	3–10 weeks	Agitation	14	*
						Insomnia	24	*
Rotella et al.	2020	All age groups	Schizophrenia or bipolar disorder	Aripiprazole: 400 mg OM Paliperidone: 78–234 mg OM / Placebo	≥ 52 weeks	Myocardial infarction	2	600
				Aripiprazole: 400 mg OM Paliperidone: 78–234 mg OM Risperidone: 25–50 mg BIW / Placebo Fluphenazine combined with haloperidol: 25.6 mg/month^a^ / Placebo plus haloperidol		Cardiac death	5	1481
Schneider-Thoma et al.	2018	All age groups (mostly aged 18–65 years, followed by children and adolescents, then older adults)	Any diagnosis (e.g. schizophrenia, bipolar disorder, major depressive disorder)	Amisulpride: 50–600 mg/d^a^ Aripiprazole: 2–882 mg/d^a^ Asenapine: 5–20 mg/d^a^ Cariprazine: 0.75–9.1 mg/d^a^ Clozapine: 24.7 or 35.8 mg/d^a^ Lurasidone: 20–160 mg/d^a^ Olanzapine: 1–16.3 mg/d^a^, 210 or 300 mg/2 weeks, or 405 mg/4 weeks Paliperidone: 1.5–337 mg/d^a^ Quetiapine: 50–800 mg/d^a^ Risperidone: 0.5–27 mg/d^a^ or 25–75 mg/2 weeks Ziprasidone: 40–130 mg/d^a^ / Placebo	1.5 hours–104 weeks	All-cause mortality	337	79,544
						Suicide mortality	333	78,853
Trinchieri et al.	2021	Mean: 82.9 years	Psychosis associated with dementia	Aripiprazole: 2–10 mg/d Haloperidol: 2 mg/d^c^ Quetiapine: 97 mg/d^c^	10 weeks	Urinary incontinence	5	1101

*Information unavailable; ^a^Mean doses; ^b^Median doses; ^c^Median of mean doses. BID, twice daily; BIW, twice weekly; *k*, number of primary studies; LAI, long-acting injection; FGA, first generation antipsychotics; SGA, second generation antipsychotics; OCD, obsessive-compulsive disorder; OM, once monthly; PTSD, post-traumatic stress disorder.

Eleven (34%) of the included articles were meta-analyses of observational studies, (Correll et al., 2022, Indrakusuma et al., 2022, Liu et al., 2021, Mortensen et al., 2020, Nosè et al., 2015, Papola et al., 2018, Seppala et al., 2018, Vancampfort et al., 2015, Wang et al., 2021, Yang et al., 2018, Zivkovic et al., 2019) reporting data on 15 adverse effects and 262 studies, with sample sizes ranging from 11,789 to over 28,000,000 (total N = 38,350,349), which compared exposed to non-exposed individuals. Primary studies were mostly case-control and cohort designs, with some self-controlled case series, cross-over or cross-sectional designs. Most reviews included non-specified or multiple diagnostic groups, and two were focused on individuals diagnosed with schizophrenia-spectrum or mood disorders. Table 2 presents the characteristics of included reviews of observational studies.

**Table 2:** Characteristics of all included meta-analyses of observational studies.

Author	Year	Population characteristics	Primary study designs	Adverse effect	Definition and determination of adverse effect	*k*	*N*
Correll et al.	2022	Schizophrenia (including first-episode and treatment-resistant schizophrenia) Age range: 10–109 years	Cohort studies	All-cause mortality	Nationwide databases used in some studies	11	*
				Suicide mortality		4	
Indrakusuma et al.	2022	Women	Case-control, nested case-control and cohort studies	Breast cancer	Medical records	5	1,232,082
Liu et al.	2021	Any diagnosis	Case-control and cohort studies	Pulmonary embolism	ICD diagnosis, autopsy records, hospital medical records, and objective tests	4	28,729,856
				Venous thromboembolism	ICD diagnosis, post-mortem records, hospital medical records, and objective tests	22	2,766,719
Mortensen et al.	2020	Mean age: 71 years	Case-control and cohort studies	Hip fracture	*	8	306,713
Nosé et al.	2015	Included studies included either age < 65 years (*k* = 2) or > 65 years (*k* = 4)	Case-control and nested case-control studies and 1 self-controlled case series	Pneumonia	ICD diagnosis, medical records, radiographical findings for aspiration pneumonia, community-acquired pneumonia, and pneumonia	6	30,659
Papola et al.	2018	Mean age: 72 years Diagnosed with dementia, Parkinson’s disease, schizophrenia, and miscellaneous conditions	Case-control and cohort studies	Fracture	ICD diagnosis, patient self-report during interviews, hospital records, and national healthcare databases and registries	7	1,753,902
Seppala et al.	2018	Age: ≥ 60 years	Case-control, cohort and cross-sectional studies	Falls	Falls and recurrent falls during follow-up or hospitalisation. Medical records, incident reports, and recall	16	*
Vancampfort et al.	2015	Mean age (range): 41 (22–73) years Diagnosed with schizophrenia or a related psychotic disorder, bipolar disorder or major depressive disorder	Cohort and cross-sectional studies	Metabolic syndrome	ATP-III, ATP-III-A, IDF or WHO diagnosis of metabolic syndrome (abdominal obesity, hyperglycaemia, hypertriglyceridemia, low high-density lipoprotein cholesterol, and hypertension)	143	13,165
Wang et al.	2021	Women exposed to antipsychotics during pregnancy	Cohort studies	Gestational diabetes mellitus	Recorded diagnosis codes, national health registers, databases, maternal report of diagnosis during interview, and recorded metformin medication prescription	6	1,876,331
Yang et al.	2018	Age range 10–100 years Any diagnosis (dementia, Alzheimer’s disease, schizophrenia, Parkinson’s disease, etc.)	Case-control and cohort studies	Cardiac death	National healthcare and patient register databases	7	409,294
				Sudden death		3	11,789
Zivkovic et al.	2019	Mean age ranged between 78 and 81 years for patients with dementia; 43–57 years for patients with other psychiatric conditions; and 45–85 years in the general population Diagnoses: dementia, schizophrenia, depression, mood disorder, or not specified	Case-control and cohort studies	Myocardial infarction	Diagnosis, hospitalisation and self-report	7	779,154
				Stroke	Hospitalisation for stroke or transient ischaemic attack, death from cerebrovascular event, and first diagnosis	13	440,685

*Information unavailable; *k* = the number of primary studies or associations; OCD, obsessive-compulsive disorder; PTSD, post-traumatic stress disorder; ATP, Adult Treatment Panel; IDF, International Diabetes Federation.

### 3.2 Quality assessment of included studies

**Fig. 1:**
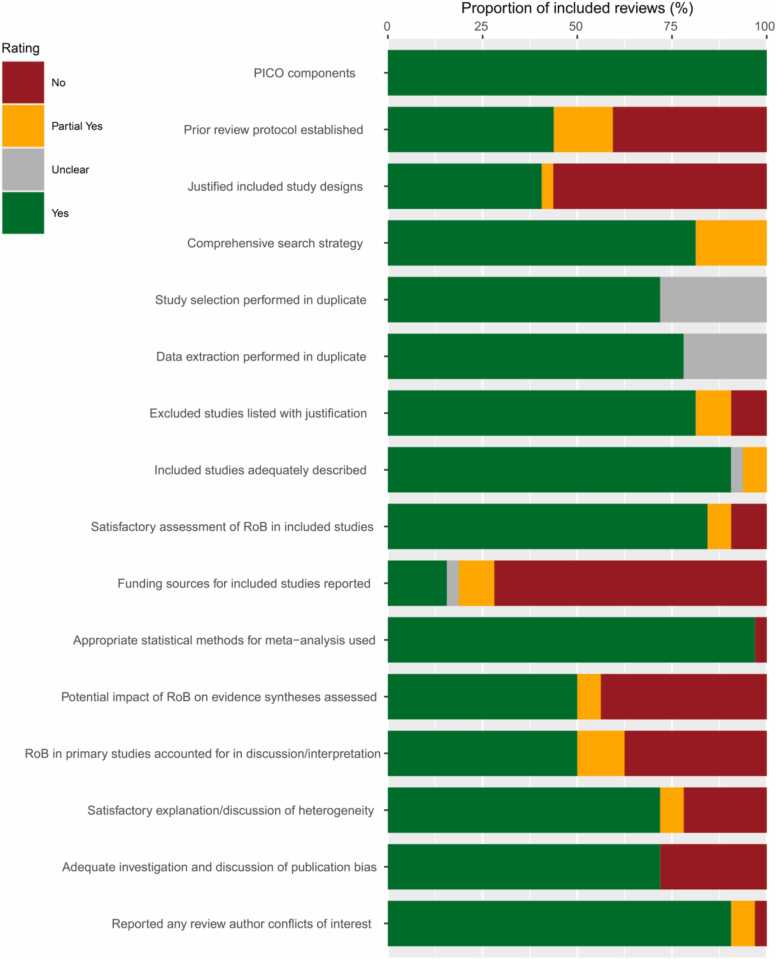
AMSTAR 2 ratings across domains for included reviews.

Of the total group of 32 included reviews, 4 (13%) were rated high quality according to AMSTAR 2, 5 (16%) were moderate, 12 (38%) were low, and 11 (34%) were critically low. For the 7 AMSTAR 2 critical domains, the lowest ratings (i.e. proportion of studies scoring “yes” in each domain) were for: 44% of included reviews explicitly established a protocol before conducting the review, 50% accounted for risk of bias in primary studies when interpreting and discussing results, and 72% adequately investigated publication bias and discussed its impact on the results of the review (see Fig. 1 for AMSTAR 2 scores across reviews).

### 3.3 Summary of adverse effects data

Included meta-analyses reported on 47 different adverse effects. In RCT meta-analyses, 38 adverse effects were reported, and in observational study meta-analyses 15 adverse effects were reported. Only 5 adverse effects (myocardial infarction, stroke, all-cause mortality, cardiac death, suicide mortality, and pneumonia) were examined in systematic reviews of both RCTs and observational studies (and for pneumonia the review of RCTs consisted of only a single trial). Table 3 summarises the effect size data across included reviews.

**Table 3:** Effect size data across adverse effect categories from included reviews of RCTs and observational studies. Cell shading corresponds to overall summary evidence score for consistency/robustness (red, < 1.5; yellow, 1.5–2.5; green, 3–4).





*Information unavailable; ^a^Derived from pooling multiple associations on review level when no sufficient primary study data were available (corresponding to e.g. individual antipsychotics, doses, diagnoses), with 95% PIs calculated from review-level heterogeneity statistics. Adverse effect categories adapted from Common Terminology Criteria for Adverse Events (CCTAE) v5.0. CI, confidence interval; I^2^, heterogeneity of the effect size estimate (for meta-analyses which reported the heterogeneity of their primary studies with the Cochran’s Q statistic, Q was converted into I^2^); PI, prediction interval; MAs, meta-analyses.

**Fig. 2:**
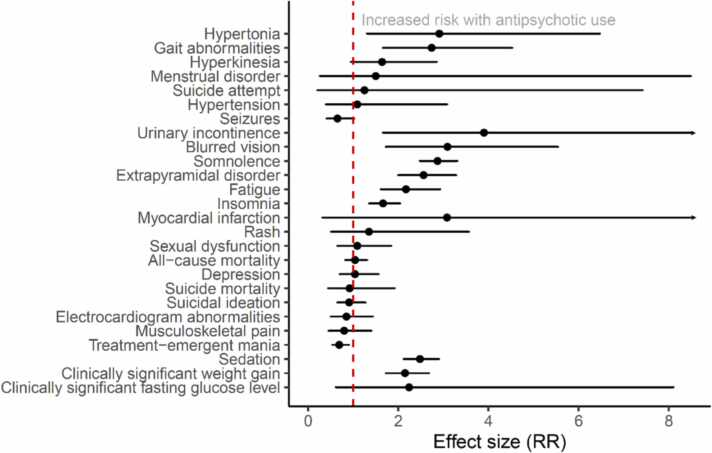
Pooled effect sizes for adverse effects with a summary quality score of 1.5 or more from meta-analyses of RCTs.

**Fig. 3:**
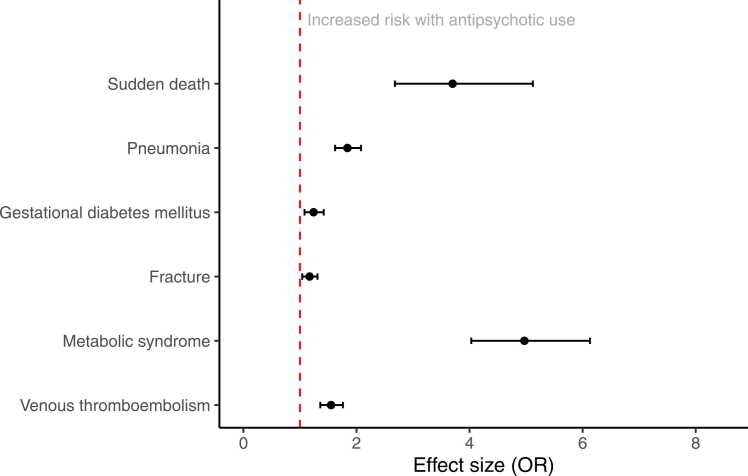
Pooled effect sizes for adverse effects with a summary quality score of 1.5 or more from meta-analyses of observational studies.

Of the 47 adverse effects, the overall score for consistency and robustness (incorporating information on prediction intervals, heterogeneity, AMSTAR 2 rating and excess significance bias) was rated as either moderate or high for 32 adverse effects (Table 4). Six of these were in observational meta-analyses and 26 in RCT meta-analyses; none had moderate/high quality scores across meta-analyses of both types of study design. Among these 32 adverse effects, the highest effect sizes were for metabolic syndrome (OR 5.0, 95% CI 4.0–6.1), urinary incontinence (RR 3.9, 95% CI 1.7–9.1), sudden death (RR 3.7, 95% CI 2.7–5.1), blurred vision (RR 3.1, 95% CI 1.7–5.5), hypertonia (RR 2.9, 95% CI 1.3–6.5), somnolence (RR 2.9, 95% CI 2.5–3.3), and gait abnormalities (RR 2.7, 95% CI 1.7–4.5) (Fig. 2, Fig. 3). We were able to calculate prediction intervals for 35 out of 38 (92%) adverse effects in RCT meta-analyses, and for 11 out of 15 (73%) adverse effects in observational study meta-analyses.

**Table 4:** Adverse effects for which summary score for consistency/robustness of effect size data was rated as moderate or high.

Adverse outcome	Study design	Effect metric	Effect size	95% CI	I^2^	95% PI excludes null?	AMSTAR 2 rating	Presence of ESS	Summary quality score
Hypertonia	RCT	RR	**2.91**	**1.31–6.47**	Low	No	High	No	3
Gait abnormalities	RCT	RR	**2.74**	**1.66–4.53**	Low	Yes	Low	No	3
Hyperkinesia	RCT	RR	1.64	0.95–2.85	Low	No	High	No	3
Menstrual disorder	RCT	RR	1.50	0.26–8.49	Low	No	High	No	3
Suicide attempt	RCT	RR	1.25	0.21–7.42	Low	No	High	No	3
Hypertension	RCT	RR	1.09	0.39–3.08	Low	No	High	No	3
Seizures	RCT	RR	0.65	0.41–1.02	Low	No	Moderate	No	2.5
Urinary incontinence	RCT	RR	**3.90**	**1.66–9.14**	Low	Yes	Critically low	Yes	2
Sudden death	Observational	RR	**3.70**	**2.68–5.12**	Low	Yes	Low	Yes	2
Blurred vision	RCT	RR	**3.09**	**1.72–5.54**	Low	Yes	Low	*	2
Somnolence	RCT	RR	**2.87**	**2.48–3.31**	Low	Yes	Critically low	Yes	2
Extrapyramidal disorder	RCT	RR	**2.56**	**2.00–3.28**	Low	No	Critically low	No	2
Fatigue	RCT	RR	**2.17**	**1.61–2.93**	Low	Yes	Low	*	2
Pneumonia	Observational	OR	**1.84**	**1.62–2.08**	Low	Yes	Low	Yes	2
Insomnia	RCT	OR	**1.66**	**1.35–2.04**	Low	Yes	Low	*	2
Gestational diabetes mellitus	Observational	RR	**1.24**	**1.08–1.42**	Low	No	Low	No	2
Fracture	Observational	OR	**1.17**	**1.04–1.31**	High	No	High	No	2
Myocardial infarction	RCT	OR	3.08	0.32–29.71	Low	No	Low	No	2
Rash	RCT	RR	1.35	0.51–3.57	Low	No	High	Yes	2
Sexual dysfunction	RCT	RR	1.09	0.65–1.84	Low	No	Critically low	No	2
All-cause mortality	RCT	RR	1.04	0.82–1.31	Low	No	High	Yes	2
Depression	RCT	RR	1.04	0.70–1.56	Low	No	Critically low	No	2
Suicide mortality	RCT	RR	0.92	0.44–1.92	Low	No	High	Yes	2
Suicidal ideation	RCT	RR	0.91	0.65–1.27	Low	No	Low	No	2
Electrocardiogram abnormalities	RCT	RR	0.85	0.51–1.43	Low	No	Critically low	No	2
Musculoskeletal pain	RCT	RR	0.80	0.46–1.40	Low	No	High	Yes	2
Treatment-emergent mania	RCT	RR	0.69	0.54–0.90	Low	*	Low	No	2
Metabolic syndrome	Observational	OR	**4.97**	**4.03–6.13**	Moderate	Yes	Critically low	*	1.5
Sedation	RCT	RR	**2.48**	**2.12–2.90**	Moderate	Yes	Low	*	1.5
Clinically significant weight gain (≥7%)	RCT	RR	**2.15**	**1.73–2.68**	Moderate	No	Critically low	No	1.5
Venous thromboembolism	Observational	OR	**1.55**	**1.36–1.76**	High	No	Moderate	No	1.5
Clinically significant fasting glucose level	RCT	RR	2.24	0.62–8.10	Low	No	Moderate	Yes	1.5

*Insufficient data available. RCT, randomised controlled trial; OR, odds ratio; RR, risk ratio; CI, confidence interval; I^2^, heterogeneity of the effect size estimate with cut-offs for low, moderate and high of 25%, 50% and 75% respectively; PI, prediction interval; ESS, excess significance bias. Lower 95% CIs for effect sizes in bold do not cross 1.

### 3.4 Circulatory system

One observational meta-analysis in people with any diagnosis reported associations with pulmonary embolism (OR 3.7, 95% CI 1.2–11.1) and venous thromboembolism (OR 1.6, 95% CI 1.4–1.8) (Liu et al., 2021) compared to those without exposure to antipsychotics. None of the four cardiovascular outcomes examined in RCTs (hypertension, hypotension, myocardial infarction and stroke) were found to be associated with antipsychotic use, though meta-analyses of observational studies reported associations for stroke and myocardial infarction.

### 3.5 Endocrinological and metabolic outcomes

One meta-analysis provided effect sizes for clinically significant weight gain in RCTs of individuals with bipolar disorder (k = 49, N = 17,167) (Bai et al., 2020). Primary study data for different antipsychotic drugs, doses and durations were pooled to produce a summary RR of 2.2 (95% CI 1.7–2.7). The largest RRs for individual antipsychotic drugs were for olanzapine 5–20 mg/day administered over 6–8 weeks (RR 36.6, 95% CI 11.7–114.0), asenapine at 10–20 mg/d administrated over 3 weeks (RR 18.0, 95% CI 2.5–131.2) and quetiapine extended release at 400–800 mg/d administered over 3 weeks (RR 18.0, 95% CI 1.1–309.3). One meta-analysis of observational studies of individuals with schizophrenia-spectrum or bipolar disorder (k = 143, N = 13,165) reported associations with metabolic syndrome (Vancampfort et al., 2015). The summary OR was the strongest association for any adverse event among observational studies (OR 5.0, 95% CI 4.0–6.1). The largest individual effect size was for clozapine (OR 7.8, 95% CI 6.0–10.2).

Second generation antipsychotics used off-licence increased the risk of endocrine abnormalities (except diabetes) in RCTs (RR 2.2, 95% CI 1.3–3.8). Among pregnant women, antipsychotic exposure was associated with the risk of gestational diabetes mellitus in observational studies (RR 1.2, 95% CI 1.1–1.4).

### 3.6 Injury

Three meta-analyses of observational studies of older adult populations (mean age 71 years, (Mortensen et al., 2020) mean age 72 years (Papola et al., 2018) and individuals ≥ 60 years old (Seppala et al., 2018)) found associations with hip fracture (OR 1.9, 95% CI 1.4–2.5), fracture (OR 1.2, 95% CI 1.0–1.3) and falls (OR 1.5, 95% CI 1.3–1.9). The OR for falls was higher when the meta-analysis was limited to primary studies conducted in the community setting (OR 2.3, 95% CI 1.2–4.3).

### 3.7 Mortality

All-cause mortality, cardiac death, and suicide mortality were examined in both RCT and observational meta-analyses. Meta-analyses of RCTs examined all-cause mortality (OR 1.0, 95% CI 0.8–1.3), cardiac death (OR 2.0, 95% CI 0.5–8.3) and suicide mortality (RR 0.9, 95% CI 0.4–1.9), but the uncertainty around the latter two estimates was large. A meta-analysis of cohort studies similarly found no association between antipsychotic use and all-cause mortality (RR 0.7, 95% CI 0.6–0.8) or suicide mortality (RR 0.7, 95% CI 0.5–1.1) among patients with schizophrenia (Correll et al., 2022). One meta-analysis of case-control and cohort studies of individuals with any diagnosis found that among antipsychotic users there were more cardiac deaths (RR 2.1, 95% CI 1.3–3.4) and sudden deaths (RR 3.7, 95% CI 2.7–5.1) than antipsychotic non-users (Yang et al., 2018).

### 3.8 Musculoskeletal and movement-related outcomes

Meta-analysis of RCTs of individuals with schizophrenia treated with paliperidone versus placebo demonstrated associations with hyperkinesia (RR 1.6, 95% CI 1.0–2.9) and hypertonia (RR 2.9, 95% CI 1.3–6.5) (Nussbaum and Stroup, 2008). The association with gait abnormalities in an RCT meta-analysis of individuals with dementia treated with second generation antipsychotics also remained significant after re-estimating the original effect size using a random-effects model (RR 2.7, 95% CI 1.7–4.5). None of these outcomes were examined in meta-analyses of observational studies.

### 3.9 Neuropsychiatric outcomes

Eleven different neuropsychiatric outcomes were considered across RCT meta-analyses, none of which were also examined in meta-analyses of observational studies. The strongest associations were with sedation (RR 2.5, 95% CI 2.1–2.9) and somnolence (RR 2.9, 95% CI 2.5–3.3). When stratified by age/population, larger RRs for sedation were generally found for older adults with dementia than adults without dementia. Across both populations, the largest individual effect sizes were for quetiapine (RR 3.7, 95% CI 2.3–6.0 for older adults with dementia and RR 2.9, 95% CI 2.6–3.2 for adults), followed by olanzapine, aripiprazole and risperidone. Significant associations (with effect sizes ranging from 1.7 to 3.6) were also found for hypersomnia in individuals with treatment-resistant depression treated with olanzapine, and for insomnia and agitation in individuals with depression, bipolar depression or mixed affective states treated with aripiprazole or cariprazine. More equivocal effects were found in meta-analyses reporting anxiety, suicidal ideation, suicide attempts, depression, treatment-emergent mania, and seizures (effect sizes ranged from 0.7 to 1.3).

### 3.10 Other outcomes

Other outcomes examined in RCT meta-analyses are summarised in Table 3. Off-label antipsychotic use was significantly associated with fatigue (RR 2.2, 95% CI 1.6–2.9). Olanzapine-fluoxetine in combination versus fluoxetine-placebo was significantly associated with peripheral oedema (reported as a pooled RR of 10). Second generation antipsychotic monotherapy and adjunctive therapy to antidepressants in major depressive disorder were significantly associated with blurred vision (RR 3.1, 95% CI 1.7–5.5). Adjunctive aripiprazole was associated with the highest risk of blurred vision (RR 4.1, 95% CI 1.7–9.8), while no significant effect was found for other antipsychotics. There was an increased risk of urinary incontinence among individuals treated with antipsychotics for psychosis associated with dementia compared to placebo (RR 3.9, 95% CI 1.7–9.1). Meta-analysis of observational studies found a significant OR for pneumonia associated with antipsychotic use in patients with all age groups (OR 1.8, 95% CI 1.6–2.1); this yielded a larger effect size for pneumonia than the re-estimated RCT effect size (OR 0.3, 95% CI 0.0–5.2). The RCT data for pneumonia was extracted from a single trial with a sample of only 517 participants. Therefore, this effect size was excluded from summary assessments. Meta-analysis of observational studies found no association between antipsychotic use and breast cancer (OR 1.1, 95% CI 0.9–1.2).

### 3.11 Subgroup analyses

Most summary effect estimates of outcomes reported in meta-analyses of observational studies had high heterogeneity. Where separate effect estimates were reported in included meta-analyses, these were examined as possible sources of heterogeneity. This was possible for classes of antipsychotics, individual antipsychotics, age, diagnosis, and observational study designs. No statistically significant differences between subgroups were found. Due to the numerous variations in pharmacological and patient variables in RCTs, no subgroup analyses were possible.

## 4 Discussion

This umbrella review systematically assessed the current state of research evidence for adverse effects associated with antipsychotic medication. We have synthesized evidence from 32 meta-analyses, which reported on over 1399 associations examined in almost 40 million participants. Umbrella review methodology was employed, including thorough assessment of the quality, consistency, and robustness of meta-analytic findings. We discuss overall conclusions on the validity and scope of the current evidence base, implications for clinical practice, and the direction of future research.

First, the overall quality of included meta-analyses was low, with 23 out of 32 included reviews rated as low or critically low according to a quality rating tool for umbrella reviews, AMSTAR 2. The weakest areas of methodology were the justification of included study designs, a priori establishment of review protocols, and sufficient consideration of risk of bias and heterogeneity. Meta-analyses without pre-registered protocols are especially prone to selective reporting (Ayorinde et al., 2020). Included meta-analyses that did account for risk of bias in primary studies used the Cochrane Risk of Bias tool, Newcastle-Ottawa Scale, and SIGN (Scottish Intercollegiate Guidelines Network) checklist. A few included meta-analyses reported high risk of selective reporting within the primary studies they included. Other sources of potential bias were inadequate matching of participant variables, funding from pharmaceutical companies, and high non-response rate.

Second, meta-analyses of RCTs and observational studies did not typically consider the same adverse effects. These different study designs are prone to specific biases, and a more complete evidence base requires information from both RCT and observational data. For example, the majority of effects examined in meta-analyses of observational studies were found to be associated with antipsychotic use, but most effect sizes had high heterogeneity, and adjustment for confounding within analyses was variable. These findings are likely therefore to be confounded. Case-control studies are particularly liable to potential selection bias, due to the difficulties of identifying a well-matched control group. Observational studies, especially of a cross-sectional design, also typically offer less strong inference of causality. Trial evidence is also prone to selection bias due to narrow inclusion criteria and restrictive recruitment processes. This can limit ecological validity or generalisability, as those entering RCTs are often less unwell than the real-world clinical population to which their findings would apply (Kennedy-Martin et al., 2015). For example, people with comorbidities or elevated suicidal risks are typically excluded from antipsychotic trials. Similarly, trial evidence is focused on short term outcomes (Blonde et al., 2018). Included reviews varied widely in this regard, and the trial period was 12 weeks or shorter in eleven included meta-analyses. While low heterogeneity was found among effect sizes extracted from RCTs, limited representativeness could underestimate heterogeneity in reported outcomes (Kravitz et al., 2004). Furthermore, adverse effects are often the secondary outcome in RCT meta-analyses, meaning their reporting may be less comprehensive compared to the primary outcome (i.e. efficacy).

Third, the differences that we found between observational and RCT meta-analyses suggest a clear role for observational studies in evidence synthesis. The clearest examples were for rarer and more severe outcomes, including mortality, MI, and stroke where the RCT evidence had wide confidence intervals. Complementing these were observational studies, where estimates were more precise owing to larger sample sizes, although the validity of conclusions may be affected by selection bias. In addition to the importance of complementarity, there were adverse effects where there was no RCT meta-analysis, including for fractures, metabolic syndrome, pulmonary emboli and venous thromboemboli, but for which observational studies provided evidence. These are important outcomes to prevent, and in the absence of RCT evidence, findings from observational designs could inform assessment and prevention. At the same time, well-powered feasible RCTs need funding.

### 4.1 Limitations

Despite the strengths of an umbrella review for summarising such a large literature, one limitation is that the defined parameters of the umbrella review (in terms of population, intervention, comparator and outcome) may not precisely match included meta-analyses. To address this, primary studies could be identified that examine relevant adverse outcomes but which are not included in the original meta-analyses. Such an undertaking was however outside the scope of the current umbrella review.

Regarding the quality assessments, AMSTAR 2 was used to assist in identifying high quality reviews, and the number of critical flaws and non-critical weaknesses was used to guide a summary of the confidence in the findings of each meta-analysis. This accepted a “partial yes” score as a positive dichotomous rating, which tends towards non-conservatism. Importantly, however, AMSTAR 2 was one aspect of how the robustness of the underlying review evidence was considered, and we tested other markers of this (including heterogeneity). The overall low quality of evidence suggests caution is warranted on the clinical interpretation of the findings. As there are many ways which quality and robustness of the evidence can be considered, and the way that the data quality is skewed, we chose to present more granular details from the underlying reviews rather than undertaking subgroup or sensitivity analyses.

In this review, we have considered the nature of the evidence for the association between antipsychotics and adverse effects in observational studies and trials. The issue of causality, and the biological underpinnings and causal pathways, is another important aspect of understanding which is not directly addressed by the current synthesis. This requires integration of other types of evidence, including experimental and imaging studies, and restricting findings to only those with these other designs and RCT evidence in support (Kaar et al., 2020).

### 4.2 Clinical recommendations

Study quality information was combined with information on prediction intervals, heterogeneity, and excess significance bias. Of the 47 adverse effects, the overall score for consistency and robustness calculated in this manner was rated as either moderate or high for 32 (68%) adverse effects. When effect sizes are considered in this context, three groups of adverse outcomes present with the most consistent evidence: endocrine and metabolic effects, movement-related outcomes (e.g. hypertonia), and sedation and sleep-related outcomes. Therefore, we would suggest these areas would be particularly relevant for clinical decision-making and discussions with patients about treatment (Achtyes et al., 2018). The review findings allow clinicians to consider the robustness and quality of the research evidence in such discussions, which will help in assisting patients to make the most informed decisions. For those adverse effects with the clearest evidence, discussion of the relative magnitude of the effect sizes summarised in this review can further add to decision-making about treatment options. Part of this discussion will also be information about patient preferences about what adverse effects are most problematic for any particular individual, and further research should gather patient views. For clinical application, it is important to note that some of the adverse effects discussed were only considered in specific described study populations (e.g. falls and fractures in studies of older adults).

A key clinical question not well covered by the included meta-analyses is absolute rates, or the magnitude of risk of adverse outcomes, given that the emphasis is on reporting of risks compared to control populations. In clinical discussion, this information would ideally be set in the context of the severity of the potential adverse outcome (e.g. mild, moderate or severe), individual preferences, and the comparative benefits on risk of relapse and other medication benefits. A less severe side effect may be more acceptable if the risk is similar to that of relapse prevention. At the same time, communicating benefits and risks will also rely on evidence synthesis of relative and absolute risks of adverse effects. This is particularly the case for communicating the risk of the most severe adverse effects, such as increased risk of sudden death which was reported by one meta-analysis of observational studies.

### 4.3 Implications for future research

This umbrella review has identified limitations to the quality of meta-analyses in the area of antipsychotic adverse effects, and future reviews should endeavour to address methodological shortfalls highlighted. Further, choice of study design and need for triangulation between study types should be considered in future reviews.

There was a clear lack of overlap between the adverse effects considered by reviews of observational and randomised studies, and further work should consider these gaps. However, both types of study design have limitations, and so other approaches should also be pursued. For example, confounding by indication and the difficulties of identifying matched control groups in observational studies can be addressed, to some extent, by using within-individual pharamco-epidemiological designs. This design investigates outcome rates in periods where the same individual is exposed or not exposed to medication, thereby controlling for time-invariant confounders.

## 5 Conclusion

We have synthesized the meta-analytic evidence from RCTs and observational studies of adverse effects of antipsychotics, and appraised the robustness of reported associations in multiple ways. Endocrine and metabolic, movement-related, and sedation/sleep-related adverse effects were clinical domains with the strongest evidence for their association with antipsychotics. Overall, however, the quality of meta-analytic evidence was low and a number of key evidence gaps remain. Future reviews should focus on adhering to methodological guidelines, consider triangulating across different study designs, and integrate information on absolute rates and relative risks to aid clinical discussion and collaborative decision-making.

## CRediT authorship contribution statement

RC completed data screening, data extraction, statistical analysis, and writing (original draft and editing). DW reviewed data extraction, and led on data visualisation, writing (original draft and editing). SF led on conceptualisation, supervision, and writing (review and editing). LF reviewed data extraction, and writing (review and editing). EO and AC writing (review and editing). All authors contributed to methodology and interpretation of results. RC is the guarantor of this review.

## Declaration of Competing Interest

None.

## Figures and Tables

**Fig. A1:**
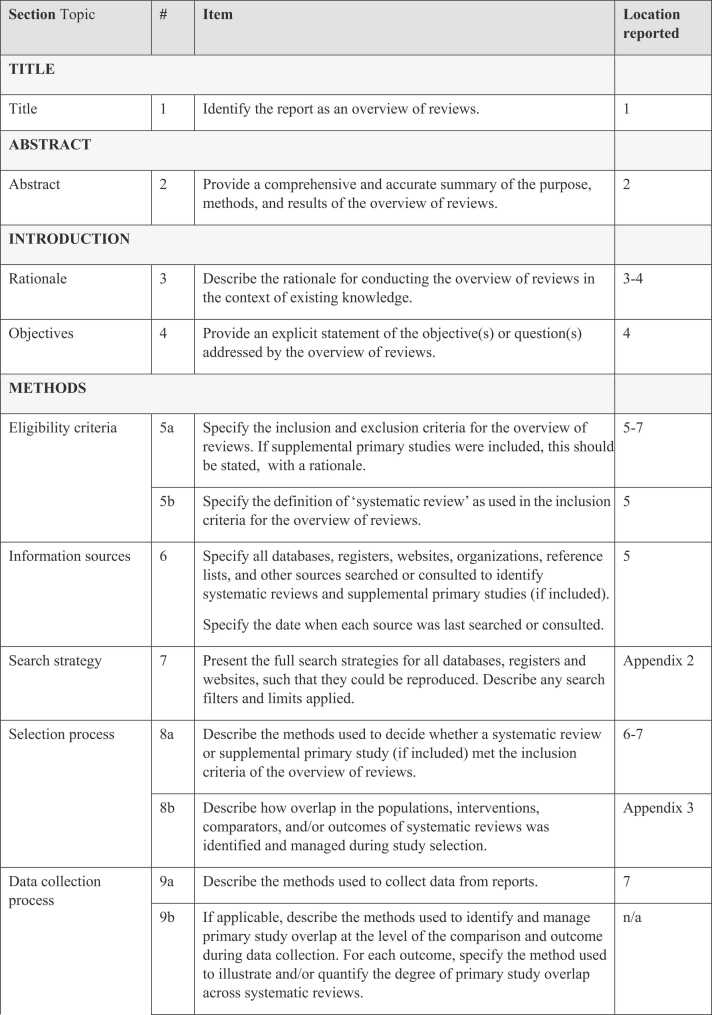

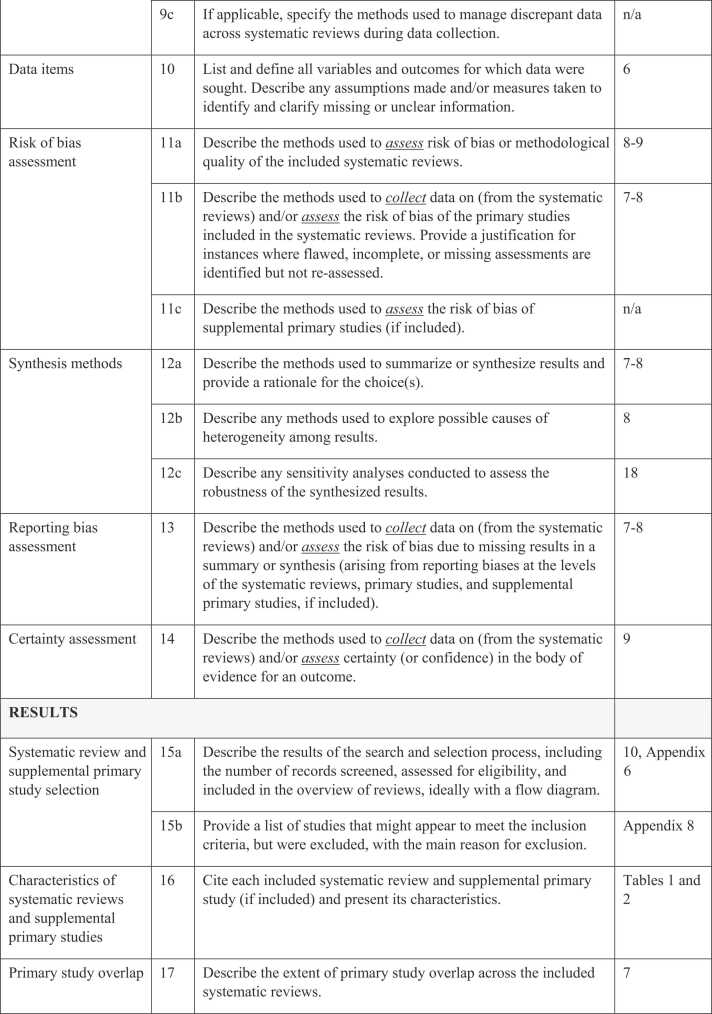

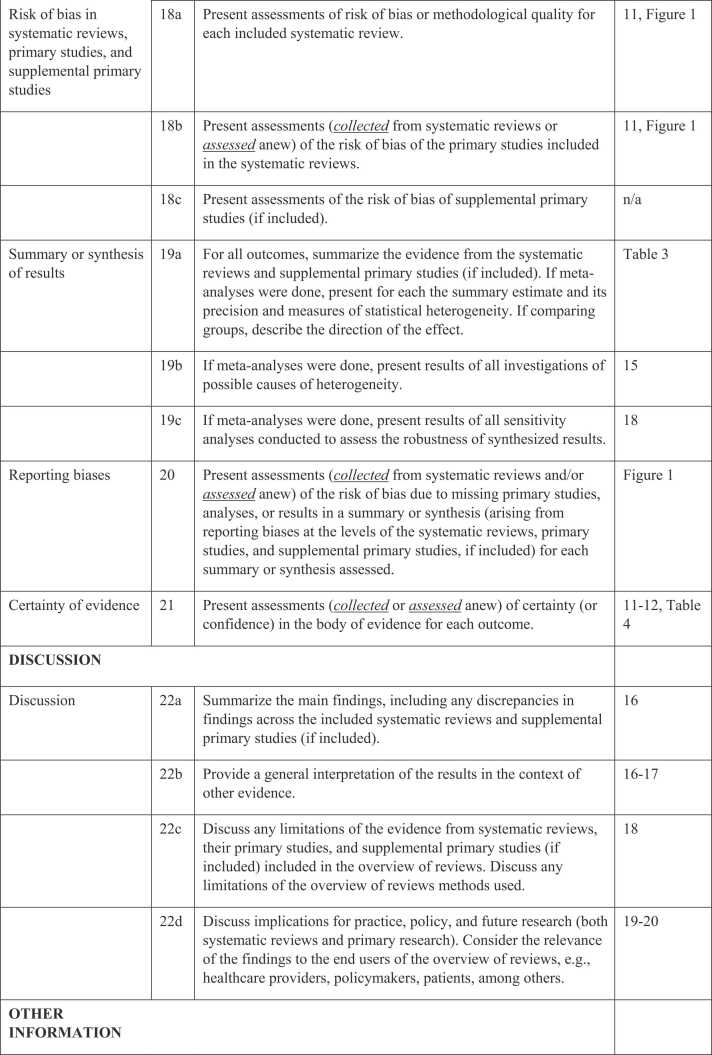

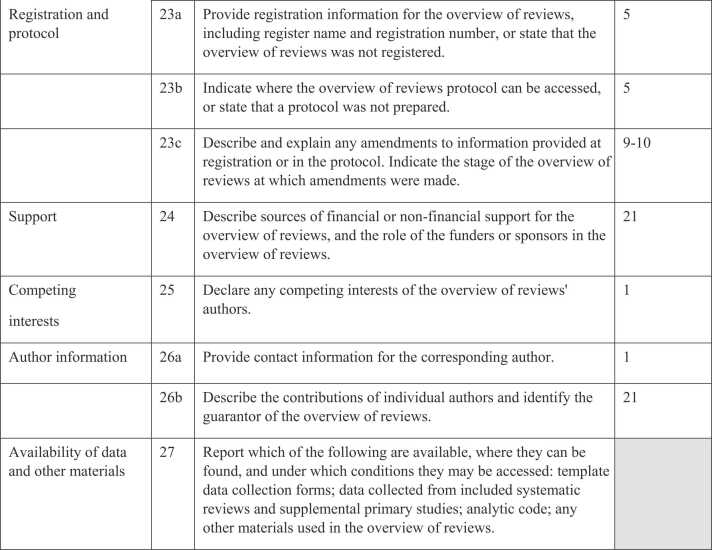
PRIOR checklist.

**Fig. A2:**
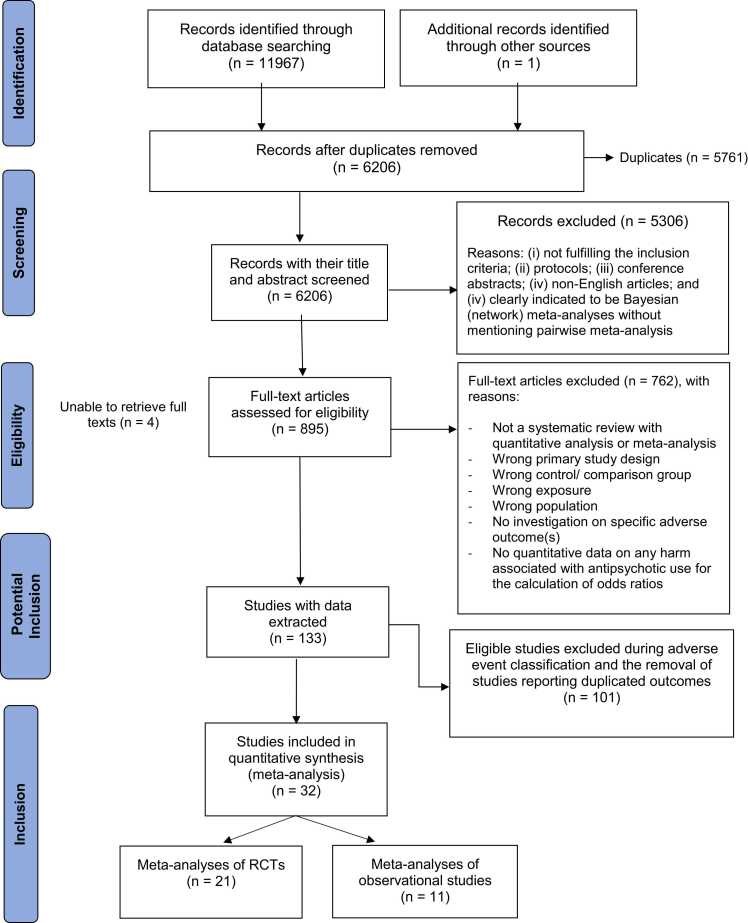
PRISMA flow chart.

**Table A1:** Excluded adverse effects with reasons.

**Adverse effect**	**Reason for exclusion**
Abdominal discomfort	Not a diagnosis - symptomatic
Abdominal pain	Not a diagnosis - symptomatic
Abdominal pain/ discomfort	Non-specific adverse effect
Abnormal thoughts	With fewer than 3 primary studies or associations
Abnormal/ blurred vision	Non-specific adverse effect
Absolute BMI change	Non-clinically significant continuous outcome
Absolute weight change	Non-clinically significant continuous outcome
Activation symptoms	Non-specific adverse effect
Acute coronary syndrome	With fewer than 3 primary studies or associations
Aggression	With fewer than 3 primary studies or associations
Agitation/ aggression	Non-specific adverse effect
Agranulocytosis	Unusable data
Akathisia-related events	Non-specific adverse effect
Anorexia	With fewer than 3 primary studies or associations
Anticholinergic events	Non-specific adverse effect
Anticholinergic symptoms	Non-specific adverse effect
Anxiety/ depression	Non-specific adverse effect
Apathy	With fewer than 3 primary studies or associations
Asthenia	Not a diagnosis - symptomatic
Asthenia/ lassitude/ increased fatigability	Non-specific adverse effect
At least one anticholinergic side effect	Non-specific adverse effect
Average change in alanine aminotransferase (ALT)	Non-clinically significant continuous outcome
Average change in albumin	Non-clinically significant continuous outcome
Average change in alkaline phosphatase	Non-clinically significant continuous outcome
Average change in aspartate amino-transferase	Non-clinically significant continuous outcome
Average change in BAR score	Non-clinically significant continuous outcome
Average change in basophils count	Non-clinically significant continuous outcome
Average change in bilirubin	Non-clinically significant continuous outcome
Average change in BMI	Non-clinically significant continuous outcome
Average change in calcium	Non-clinically significant continuous outcome
Average change in creatine kinase level	Non-clinically significant continuous outcome
Average change in creatine phosphokinase	Non-clinically significant continuous outcome
Average change in diastolic blood pressure	Non-clinically significant continuous outcome
Average change in erythrocyte count	Non-clinically significant continuous outcome
Average change in fasting glucose	Non-clinically significant continuous outcome
Average change in fasting triglycerides	Non-clinically significant continuous outcome
Average change in gamma-glutamyl transferase	Non-clinically significant continuous outcome
Average change in glucose level	Non-clinically significant continuous outcome
Average change in haemoglobin count	Non-clinically significant continuous outcome
Average change in high lipoprotein cholesterol	Non-clinically significant continuous outcome
Average change in insulin serum level	Non-clinically significant continuous outcome
Average change in leukocyte count	Non-clinically significant continuous outcome
Average change in low lipoprotein cholesterol	Non-clinically significant continuous outcome
Average change in mean cell haemoglobin concentration	Non-clinically significant continuous outcome
Average change in monocytes count	Non-clinically significant continuous outcome
Average change in platelet count	Non-clinically significant continuous outcome
Average change in prolacin	Non-clinically significant continuous outcome
Average change in pulse rate	Non-clinically significant continuous outcome
Average change in QTc interval	Continuous outcome
Average change in SAS score	Non-clinically significant continuous outcome
Average change in score on AIMS (Abnormal Involuntary Movements Scale)	Non-clinically significant continuous outcome
Average change in score on BARS (Barnes Akathisia Rating Scale)	Non-clinically significant continuous outcome
Average change in score on SAS (Simpson-Angus Scale)	Non-clinically significant continuous outcome
Average change in segmented neutrophils count	Non-clinically significant continuous outcome
Average change in serum level	Non-clinically significant continuous outcome
Average change in sitting diastolic blood pressure	Non-clinically significant continuous outcome
Average change in sitting pulse rate	Non-clinically significant continuous outcome
Average change in sitting systolic blood pressure	Non-clinically significant continuous outcome
Average change in standing diastolic blood pressure	Non-clinically significant continuous outcome
Average change in standing pulse rate	Non-clinically significant continuous outcome
Average change in standing systolic blood pressure	Non-clinically significant continuous outcome
Average change in supine diastolic blood pressure	Non-clinically significant continuous outcome
Average change in supine pulse rate	Non-clinically significant continuous outcome
Average change in supine systolic blood pressure	Non-clinically significant continuous outcome
Average change in systolic blood pressure	Non-clinically significant continuous outcome
Average change in total cholesterol level	Non-clinically significant continuous outcome
Average change in triglyceride level	Non-clinically significant continuous outcome
Average change in urea nitrogen	Non-clinically significant continuous outcome
Average change in waist circumference	Non-clinically significant continuous outcome
Average change in weight	Non-clinically significant continuous outcome
Average total sitting pulse score	Non-clinically significant continuous outcome
Average total standing pulse score	Non-clinically significant continuous outcome
Average weight gain	Non-clinically significant continuous outcome
Blood glucose abnormalities	Non-specific - individual outcomes within AE already included
Body pain	Not a diagnosis - symptomatic
Bradycardia	Not a diagnosis - symptomatic
Bruise	Not a diagnosis - symptomatic
Cardiomyopathty	Unusable data
Cardiovascular events	Non-specific adverse effect
Cardiovasular-other	Non-specific adverse effect
Cerebrovascular events	Non-specific adverse effect
Change in alanine transaminase	Non-clinically significant continuous outcome
Change in appetite	Non-clinically significant continuous outcome
Change in aspartate transaminase	Non-clinically significant continuous outcome
Change in basophils count	Non-clinically significant continuous outcome
Change in blood calcium	Non-clinically significant continuous outcome
Change in BMI (kg/m^2)	Non-clinically significant continuous outcome
Change in cholesterol	Non-clinically significant continuous outcome
Change in fasting glucose	Non-clinically significant continuous outcome
Change in fasting glycerides	Non-clinically significant continuous outcome
Change in high-density lipoprotein cholesterol	Non-clinically significant continuous outcome
Change in leukocytes count	Non-clinically significant continuous outcome
Change in low-density lipoprotein cholesterol	Non-clinically significant continuous outcome
Change in monocytes count	Non-clinically significant continuous outcome
Change in prolactin level	Non-clinically significant continuous outcome
Change in QTc interval	Non-clinically significant continuous outcome
Change in score on AIMS (Abnormal Involuntary Movements Scale)	Non-clinically significant continuous outcome
Change in score on BARS (Barnes Akathisia Rating Scale)	Non-clinically significant continuous outcome
Change in score on SAS (Simpson-Angus Scale)	Non-clinically significant continuous outcome
Change in segmented neutrophils count	Non-clinically significant continuous outcome
Change in total cholesterol	Non-clinically significant continuous outcome
Change in triglycerides	Non-clinically significant continuous outcome
Change in triglycerides level	Non-clinically significant continuous outcome
Change in γ-glutamyl transferase	Non-clinically significant continuous outcome
Clinically significant alanine aminotransferase level	With fewer than 3 primary studies or associations
Clinically significant change in alanine transferase	With fewer than 3 primary studies or associations
Clinically significant fasting triglyceride level	With fewer than 3 primary studies or associations
Clinically significant HbA1C levels	With fewer than 3 primary studies or associations
Clinically significant increase in alanine transferase	With fewer than 3 primary studies or associations
Clinically significant weight loss	With fewer than 3 primary studies or associations
Cold/ flu symptoms	Not a diagnosis - symptomatic
Concentration difficulties (≥moderate severity and ≥5%)	With fewer than 3 primary studies or associations
Constipation	Not a diagnosis - symptomatic
Cough	Not a diagnosis - symptomatic
Decreased appetite	Not a diagnosis - symptomatic
Decreased duration of sleep	Not a diagnosis - symptomatic
Decreased high-density lipoprotein	Non-clinically significant
Decreased libido	Not a diagnosis - symptomatic
Deliberate self-injury or violent behaviour	Non-specific adverse effect
Diarrhoea	Not a diagnosis - symptomatic
Disturbance in attention	With fewer than 3 primary studies or associations
Dizziness	Not a diagnosis - symptomatic
Drowsiness/ somnolence	Non-specific adverse effect
Dysarthria	With fewer than 3 primary studies or associations
Dyskinetic movements total score	Continuous outcome
Dyspepsia	Not a diagnosis - symptomatic
Earache	Not a diagnosis - symptomatic
Edema	Not a diagnosis - symptomatic
Epistaxis (nosebleed)	Not a diagnosis - symptomatic
EPS scale	Continuous outcome
Excessive sweating	Not a diagnosis - symptomatic
Failing memory	With fewer than 3 primary studies or associations
Forgetful or confusion	With fewer than 3 primary studies or associations
Galactorrhoea	Not a diagnosis - symptomatic
Gastroenteritis	Not a diagnosis - symptomatic
Gynaecomastia	Not a diagnosis - symptomatic
Headache	Not a diagnosis - symptomatic
High triglycerides	With fewer than 3 primary studies or associations
Hypersalivation (drooling)	Not a diagnosis - symptomatic
Increase of ≥ 15 beats/ min	With fewer than 3 primary studies or associations
Increased appetite	Not a diagnosis - symptomatic
Increased appetite/ weight gain	Non-specific adverse effect
Increased dream activity	Not a diagnosis - symptomatic
Increased duration of sleep	Not a diagnosis - symptomatic
Increased fasting glucose	Non-clinically significant
Increased fasting triglycerides	Non-clinically significant
Increased low-density lipoprotein	Non-clinically significant
Increased tendency to sweating	Not a diagnosis - symptomatic
Infection	Not a diagnosis - symptomatic
Influenza-like symptoms	Not a diagnosis - symptomatic
Injection pain	Not a diagnosis - symptomatic
Irritability	Non-specific adverse effect
Joint disorder	With fewer than 3 primary studies or associations
Liver function test abnormalities	With fewer than 3 primary studies or associations
Manic switching	With fewer than 3 primary studies or associations
Mastalgia	Not a diagnosis - symptomatic
Metabolic lab abnormalities	Non-specific - individual outcomes within AE already included
Muscle rigidity	With fewer than 3 primary studies or associations
Myalgia	With fewer than 3 primary studies or associations
Myocarditis	Unusable data
Nasal congestion	Not a diagnosis - symptomatic
Nasopharyngitis	Not a diagnosis - symptomatic
Nausea	Not a diagnosis - symptomatic
Nausea/ vomiting	Non-specific adverse effect
Nervousness	Not a diagnosis - symptomatic
Neurological events	Non-specific adverse effect
Neutropenia at a threshold of < 1000	Unusable data
Neutropenia at a threshold of < 1500	Unusable data
Neutropenia at a threshold of < 500	Unusable data
Oral hypoesthesia	Not a diagnosis - symptomatic
Pain in extremity	Not a diagnosis - symptomatic
Palpitation/ tachycardia	Non-specific adverse effect
Palpitations	Not a diagnosis - symptomatic
Parkinsonism total score	Continuous outcome
Polyuria/ polydipsia	With fewer than 3 primary studies or associations
Potential prolactin-related AEs	Non-specific adverse effect
Prolactin increase	Non-clinically significant continuous outcome
Prolactin increase-associated effects	Non-specific adverse effect
Prolactin-related AEs	Non-specific adverse effect
Pruritis	Not a diagnosis - symptomatic
Psychomotor hyperactivity	With fewer than 3 primary studies or associations
Pyrexia (fever)	Not a diagnosis - symptomatic
Receiving medications for extrapyramidal AEs	Not a diagnosis
Respiratory tract infection/ inflammation (rhinitis/ pharyngitis/ nasopharyngitis)	Non-specific adverse effect
Restlessness	Not a diagnosis - symptomatic
Rhinitis	Not a diagnosis - symptomatic
Rhinorrhea	Not a diagnosis - symptomatic
Salivation	Not a diagnosis - symptomatic
Sedation-related events	Non-specific adverse effect
Sedation/ somnolence	Non-specific adverse effect
Shift from < 3 to ≥ 3 metabolic risk factors	With fewer than 3 primary studies or associations
Sinus tachycardia	Not a diagnosis - symptomatic
Skin irritation/ rash	Non-specific adverse effect
Sleep problems	Non-specific adverse effect
Sleepiness/ sedation	Non-specific adverse effect
Somnolence/sedation	Non-specific adverse effect
Sore throat	Not a diagnosis - symptomatic
Stomachache/ abdominal pain	Non-specific adverse effect
Suicide-related adverse events	Non-specific adverse effect
Suicide-related behaviours	Non-specific adverse effect
Sweating	Not a diagnosis - symptomatic
Tachycardia	Not a diagnosis - symptomatic
Tension	With fewer than 3 primary studies or associations
Thirst	Not a diagnosis - symptomatic
Toothache	Not a diagnosis - symptomatic
Toxicity	With fewer than 3 primary studies or associations
Treatment-emergent psychiatric disorder	Non-specific adverse effect
Uneasy feeling	With fewer than 3 primary studies or associations
Upper respiratory infection	Not a diagnosis - symptomatic
Urinary symptoms	Non-specific adverse effect
Use of anti-EPS medication	Not a diagnosis
Use of anticholinergic medication	Not a diagnosis
Use of antiparkinson medication	Not a diagnosis
Use of anxiolytic medication	Not a diagnosis
Use of benzodiazepines	Not a diagnosis
Use of beta-blockers medication	Not a diagnosis
Viral gastroenteritis	Not a diagnosis - symptomatic
Vomiting	Not a diagnosis - symptomatic
Weight change	Non-clinically significant
Weight gain	Non-clinically significant
Weight loss	Non-clinically significant
Worsening psychotic symptoms	With fewer than 3 primary studies or associations
Xerostomia (dry mouth)	Not a diagnosis - symptomatic

**Table A 2:** Eligible but excluded reviews with reasons.

**Eligible but excluded studies**	**Reasons for exclusion**
Arasteh O, Nomani H, Baharara H, Sadjadi SA, Mohammadpour AH, Ghavami V, et al. Antipsychotic drugs and risk of developing venous thromboembolism and pulmonary embolism: A systematic review and meta-analysis. *Curr Vasc Pharmacol* 2020; **18**(6): 632–43.	Overlapping observational study meta-analysis: not the most recently published
Arbaizar B, Dierssen-Sotos T, Gómez-Acebo I, Llorca J. Aripiprazole in major depression and mania: meta-analyses of randomized placebo-controlled trials. *Gen Hosp Psychiatry* 2009; **31**(5): 478–83.	Overlapping RCT meta-analysis and reported other outcomes excluded from our analysis
Ardizzone I, Nardecchia F, Marconi A, Carratelli TI, Ferrara M. Antipsychotic medication in adolescents suffering from Schizophrenia: A meta-analysis of randomized controlled trials. *Psychopharmacol Bull* 2010; **43**(2): 45–66.	Overlapping RCT meta-analysis
Barbui C, Conti V, Cipriani A. Antipsychotic drug exposure and risk of venous thromboembolism: a systematic review and meta-analysis of observational studies. *Drug Saf* 2014; **37**(2): 79–90.	Overlapping observational study meta-analysis: not the most recently published
Barton BB, Segger F, Fischer K, Obermeier M, Musil R. Update on weight-gain caused by antipsychotics: a systematic review and meta-analysis. *Expert Opin Drug Saf* 2020; **19**(3): 295–314.	Overlapping RCT meta-analysis and reported other outcomes excluded from our analysis
Bosnjak Kuharic D, Kekin I, Hew J, Rojnic Kuzman M, Puljak L. Interventions for prodromal stage of psychosis. *Cochrane Database Syst Rev* 2019; (11).	Overlapping RCT meta-analysis and reported other outcomes excluded from our analysis
Cai L, Chen G, Yang H, Bai Y. Efficacy and safety profiles of mood stabilizers and antipsychotics for bipolar depression: a systematic review. *Int Clin Psychopharmacol* 2023; **38**(4): 249–60.	Overlapping RCT meta-analysis and reported other outcomes excluded from our analysis
Campforts B, Drukker M, Crins J, van Amelsvoort T, Bak M. Association between antipsychotic medication and clinically relevant weight change: Meta-analysis. *BJPsych Open* 2023; **9**.	Overlapping RCT meta-analysis
Ceraso A, Lin JJ, Schneider-Thoma J, Siafis S, Heres S, Kissling W, et al. Maintenance Treatment With Antipsychotic Drugs in Schizophrenia: A Cochrane Systematic Review and Meta-analysis. *Schizophr Bull* 2022; **48**(4): 738–40.	Overlapping RCT meta-analysis and reported other outcomes excluded from our analysis
Ceraso A, Lin JJ, Schneider-Thoma J, Siafis S, Tardy M, Komossa K, et al. Maintenance treatment with antipsychotic drugs for schizophrenia. *Cochrane Database Syst Rev* 2020; **8**(8): CD008016.	Overlapping RCT meta-analysis
Chang HY, Tseng PT, Stubbs B, Chu CS, Li DJ, Fornaro M, et al. The efficacy and tolerability of paliperidone in mania of bipolar disorder: A preliminary meta-analysis. *Exp Clin Psychopharmacol* 2017; **25**(5): 422–33.	Overlapping RCT meta-analysis and reported other outcomes excluded from our analysis
Chung AK, Chua SE. Effects on prolongation of Bazett's corrected QT interval of seven second-generation antipsychotics in the treatment of schizophrenia: a meta-analysis. *Journal of Psychopharmacology* 2011; **25**(5): 646–66.	Overlapping RCT meta-analysis and reported other outcomes excluded from our analysis
Cipriani A, Rendell JM, Geddes J. Olanzapine in long-term treatment for bipolar disorder. *Cochrane Database Syst Rev* 2009; (1): CD004367.	Overlapping RCT meta-analysis and reported other outcomes excluded from our analysis
Citrome L. Cariprazine for bipolar depression: What is the number needed to treat, number needed to harm and likelihood to be helped or harmed? *Int J Clin Pract* 2019; **73**(10): e13397.	Overlapping RCT meta-analysis and reported other outcomes excluded from our analysis
Cooper H, Mishriky R, Reyad AA. Efficacy and safety of cariprazine in acute management of psychiatric disorders: A meta-analysis of randomized controlled trials. *Psychiatr Danub* 2020; **32**(1): 36–45.	Overlapping RCT meta-analysis and reported other outcomes excluded from our analysis
Davidson M WM,; Soares K.;. Novel antipsychotics in the treatment of psychosis and aggression associated with dementia: A meta-analysis of randomized controlled clinical trials: Discussion 5. *Int Psychogeriatr* 2000; **12**(SUPPL. 1): 279–80.	Overlapping RCT meta-analysis
De Fruyt J, Deschepper E, Audenaert K, Constant E, Floris M, Pitchot W, et al. Second generation antipsychotics in the treatment of bipolar depression: a systematic review and meta-analysis. *Journal of Psychopharmacology* 2012; **26**(5): 603–17.	Overlapping RCT meta-analysis: included less primary studies than the selected meta-analysis
De Hert M, Yu W, Detraux J, Sweers K, Van Winkel R, Correll CU. Body weight and metabolic adverse effects of asenapine, iloperidone, lurasidone and paliperidone in the treatment of schizophrenia and bipolar disorder: A systematic review and exploratory meta-analysis. *CNS Drugs* 2012; **26**(9): 733–59.	Overlapping RCT meta-analysis and reported other outcomes excluded from our analysis
Deb S, Roy M, Limbu B, Akrout Brizard B, Murugan M, Roy A, et al. Randomised controlled trials of antipsychotics for people with autism spectrum disorder: a systematic review and a meta-analysis. *Psychol Med* 2023: 1–9.	Overlapping RCT meta-analysis and reported other outcomes excluded from our analysis
Depping AM, Komossa K, Kissling W, Leucht S. Second-generation antipsychotics for anxiety disorders. *Cochrane Database Syst Rev* 2010: (12): CD008120.	Overlapping RCT meta-analysis and reported other outcomes excluded from our analysis
Di X, Chen M, Shen S, Cui X. Antipsychotic use and Risk of Venous Thromboembolism: A Meta-Analysis. *Psychiatry Res* 2021; **296**: 113691.	Overlapping observational study meta-analysis: sample smaller than the selected meta-analysis
Domecq JP, Prutsky G, Leppin A, Sonbol MB, Altayar O, Undavalli C, et al. Clinical review: Drugs commonly associated with weight change: a systematic review and meta-analysis. *J Clin Endocr* 2015; **100**(2): 363–70.	The reported adverse outcomes were excluded from our analysis
Douglas-Hall P, Curran S, Bird V, Taylor D. Aripiprazole: A Review of its Use in the Treatment of Irritability Associated with Autistic Disorder Patients Aged 6–17. *Journal of Central Nervous System Disease* 2011; (3): 143–53.	Overlapping RCT meta-analysis and reported other outcomes excluded from our analysis
Fang F, Sun H, Wang Z, Ren M, Calabrese JR, Gao K. Antipsychotic Drug-Induced Somnolence: Incidence, Mechanisms, and Management. *CNS Drugs* 2016; **30**(9): 845–67.	Overlapping RCT meta-analysis
Fang F, Wang Z, Wu R, Calabrese JR, Gao K. Is there a 'weight neutral' second-generation antipsychotic for bipolar disorder? *Expert Rev Neurother* 2017; **17**(4): 407–18.	Overlapping RCT meta-analysis and reported other outcomes excluded from our analysis
Flank J, Sung L, Dvorak CC, Spettigue W, Dupuis LL. The safety of olanzapine in young children: a systematic review and meta-analysis. *Drug Saf* 2014; **37**(10): 791–804.	Overlapping RCT meta-analysis and reported other outcomes excluded from our analysis
Fung LK, Mahajan R, Nozzolillo A, Bernal P, Krasner A, Jo B, et al. Pharmacologic Treatment of Severe Irritability and Problem Behaviors in Autism: A Systematic Review and Meta-analysis. *Pediatrics* 2016; **137**: S124-S35.	Overlapping RCT meta-analysis and reported other outcomes excluded from our analysis
Fusar-Poli P, Kempton MJ, Rosenheck RA. Efficacy and safety of second-generation long-acting injections in schizophrenia: A meta-analysis of randomized-controlled trials. *Int Clin Psychopharmacol* 2013; **28**(2): 57–66.	Overlapping RCT meta-analysis and reported other outcomes excluded from our analysis
Gao K, Ganocy SJ, Gajwani P, Muzina DJ, Kemp DE, Calabrese JR. A review of sensitivity and tolerability of antipsychotics in patients with bipolar disorder or schizophrenia: focus on somnolence. *J Clin Psychiatry* 2008; **69**(2): 302–9.	Overlapping RCT meta-analysis
Gao K, Kemp DE, Ganocy SJ, Gajwani P, Xia G, Calabrese JR. Antipsychotic-induced extrapyramidal side effects in bipolar disorder and schizophrenia: A systematic review. *J Clin Psychopharmacol* 2008; **28**(2): 203–9.	Overlapping RCT meta-analysis and reported other outcomes excluded from our analysis
Harrington CA, English C. Tolerability of paliperidone: A meta-analysis of randomized, controlled trials. *Int Clin Psychopharmacol* 2010; **25**(6): 334–41.	Overlapping RCT meta-analysis
Hirsch LE, Pringsheim T. Aripiprazole for autism spectrum disorders (ASD). *Cochrane Database Syst Rev* 2016; (6): CD009043.	Overlapping RCT meta-analysis and reported other outcomes excluded from our analysis
Hoffman J, Williams T, Rothbart R, Ipser JC, Fineberg N, Chamberlain SR, et al. Pharmacotherapy for trichotillomania. *Cochrane Database Syst Rev* 2021; **9**(9): CD007662.	Overlapping RCT meta-analysis and reported other outcomes excluded from our analysis
Hollis C, Pennant M, Cuenca J, Glazebrook C, Kendall T, Whittington C, et al. Clinical effectiveness and patient perspectives of different treatment strategies for tics in children and adolescents with tourette syndrome: A systematic review and qualitative analysis. *Health Technol Assess* 2016; **20**(4): 1-viii.	Overlapping RCT meta-analysis and reported other outcomes excluded from our analysis
Hsu W-T, Esmaily-Fard A, Lai C-C, Zala D, Lee S-H, Chang S-S, et al. Antipsychotics and the Risk of Cerebrovascular Accident: A Systematic Review and Meta-Analysis of Observational Studies. *J Am Med Dir Assoc* 2017; **18**(8): 692–9.	Overlapping observational study meta-analysis: not the most recently published
Huang KL, Fang CJ, Hsu CC, Wu SI, Juang JJ, Stewart R. Myocardial infarction risk and antipsychotics use revisited: a meta-analysis of 10 observational studies. *J Psychopharmacol* 2017; **31**(12): 1544–55.	Overlapping observational study meta-analysis: not the most recently published
Huhn M, Arndt T, Schneider-Thoma J, Leucht S. Effects of antipsychotics on heart rate in treatment of schizophrenia: a systematic review and meta-analysis. *Ther Adv Psychopharmacol* 2022; **12**: 20451253221097261.	The reported adverse outcomes were excluded from our analysis
Jia N, Li Z, Li X, Jin M, Liu Y, Cui X, et al. Long-term effects of antipsychotics on mortality in patients with schizophrenia: a systematic review and meta-analysis. *Braz J Psychiatry* 2022; **44**(6): 664–73.	The reported adverse outcome was excluded from our analysis
Kishi T, Matsuda Y, Iwata N, Correll CU. Antipsychotics for cocaine or psychostimulant dependence: Systematic review and meta-analysis of randomized, placebo-controlled trials. *J Clin Psychiatry* 2013; **74**(12): e1169-e80.	Overlapping RCT meta-analysis
Kishi T, Sevy S, Chekuri R, Correll CU. Antipsychotics for primary alcohol dependence: A systematic review and meta-analysis of placebo-controlled trials. *J Clin Psychiatry* 2013; **74**(7): e642-e54.	Overlapping RCT meta-analysis and reported other outcomes excluded from our analysis
Kishi T, Matsunaga S, Iwata N. Mortality Risk Associated with Long-acting Injectable Antipsychotics: A Systematic Review and Meta-analyses of Randomized Controlled Trials. *Schizophr Bull* 2016; **42**(6): 1438–45.	Overlapping RCT meta-analysis
Kishi T, Oya K, Iwata N. Long-acting injectable antipsychotics for prevention of relapse in bipolar disorder: A systematic review and meta-analyses of randomized controlled trials. *Int J Neuropsychopharmacol* 2016; **19**(9): 1–10.	Overlapping RCT meta-analysis and reported other outcomes excluded from our analysis
Kishi T, Nakamura H, Iwata N. Differences in the incidence of lurasidone adverse events between depressive disorders and schizophrenia in double-blind, randomized, placebo-controlled trials: a meta-analysis. *Psychopharmacology (Berl)* 2021; **238**(12): 3585–93.	Overlapping RCT meta-analysis and reported other outcomes excluded from our analysis
Kishi T, Sakuma K, Okuya M, Matsuda Y, Esumi S, Hashimoto Y, et al. Effects of a conventional mood stabilizer alone or in combination with second-generation antipsychotics on recurrence rate and discontinuation rate in bipolar I disorder in the maintenance phase: A systematic review and meta-analysis of randomized, placebo-controlled trials. *Bipolar Disord* 2021; **23**(8): 789–800.	Overlapping RCT meta-analysis and reported other outcomes excluded from our analysis
Kishi T, Sakuma K, Iwata N. Paliperidone palmitate vs. paliperidone extended-release for the acute treatment of adults with schizophrenia: a systematic review and pairwise and network meta-analysis. *Transl Psychiatry* 2022; **12**(1): 519.	Overlapping RCT meta-analysis and reported other outcomes excluded from our analysis
Komossa K, Depping AM, Gaudchau A, Kissling W, Leucht S. Second-generation antipsychotics for major depressive disorder and dysthymia. *Cochrane Database Syst Rev* 2010; (12): CD008121.	Overlapping RCT meta-analysis and reported other outcomes excluded from our analysis
Komossa K, Depping AM, Meyer M, Kissling W, Leucht S. Second‐generation antipsychotics for obsessive compulsive disorder. *Cochrane Database Syst Rev* 2010; (12): CD008141.	Overlapping RCT meta-analysis and reported other outcomes excluded from our analysis
Krause M, Huhn M, Schneider-Thoma J, Rothe P, Smith RC, Leucht S. Antipsychotic drugs for elderly patients with schizophrenia: A systematic review and meta-analysis. *Eur Neuropsychopharmacol* 2018; **28**(12): 1360–70.	Overlapping RCT meta-analysis and reported other outcomes excluded from our analysis
Krause M, Zhu Y, Huhn M, Schneider-Thoma J, Bighelli I, Chaimani A, et al. Efficacy, acceptability, and tolerability of antipsychotics in children and adolescents with schizophrenia: A network meta-analysis. *Eur Neuropsychopharmacol* 2018; **28**(6): 659–74.	Overlapping RCT meta-analysis and reported other outcomes excluded from our analysis
Kucukgoncu S, Guloksuz S, Celik K, Bahtiyar MO, Luykx JJ, Rutten BPF, et al. Antipsychotic Exposure in Pregnancy and the Risk of Gestational Diabetes: A Systematic Review and Meta-analysis. *Schizophr Bull* 2020; **46**(2): 311–8.	Overlapping observational study meta-analysis: not the most recently published
Leucht S, Arbter D, Engel RR, Kissling W, Davis JM. How effective are second-generation antipsychotic drugs? A meta-analysis of placebo-controlled trials. *Molecular Psychiatry* 2009; **14**(4): 429–47.	Overlapping RCT meta-analysis and reported other outcomes excluded from our analysis
Leung JCN, Ng DWY, Chu RYK, Chan EWW, Huang L, Lum DH, et al. Association of antipsychotic use with breast cancer: a systematic review and meta-analysis of observational studies with over 2 million individuals. *Epidemiol Psychiatr Sci* 2022; **31**: e61.	Overlapping observational study meta-analysis: included more primary studies than the selected meta-analysis but reported outcome in HR instead of OR
Li DJ, Tseng PT, Stubbs B, Chu CS, Chang HY, Vieta E, et al. Efficacy, safety and tolerability of aripiprazole in bipolar disorder: An updated systematic review and meta-analysis of randomized controlled trials. *Prog Neuropsychopharmacol Biol Psychiatry* 2017; **79**(Pt B): 289–301.	Overlapping RCT meta-analysis and reported other outcomes excluded from our analysis
Li XH, Zhong XM, Lu L, Zheng W, Wang SB, Rao WW, et al. The prevalence of agranulocytosis and related death in clozapine-treated patients: a comprehensive meta-analysis of observational studies. *Psychol Med* 2020; **50**(4): 583–94.	Unable to calculate OR or RR for reported outcomes
Lieb K, Vollm B, Rucker G, Timmer A, Stoffers JM. Pharmacotherapy for borderline personality disorder: Cochrane systematic review of randomised trials. *BJPsych* 2010; **196**(1): 4–12.	Overlapping RCT meta-analysis and reported other outcomes excluded from our analysis
Luan S, Wan H, Zhang L, Zhao H. Efficacy, acceptability, and safety of adjunctive aripiprazole in treatment-resistant depression: A meta-analysis of randomized controlled trials. *Neuropsychiatr Dis Treat* 2018; **14**: 467–77.	Overlapping RCT meta-analysis and reported other outcomes excluded from our analysis
Maayan N, Quraishi SN, David A, Jayaswal A, Eisenbruch M, Rathbone J, et al. Fluphenazine decanoate (depot) and enanthate for schizophrenia. *Cochrane Database Syst Rev* 2015; (2).	Overlapping RCT meta-analysis and reported other outcomes excluded from our analysis
Majer IM, Gaughran F, Sapin C, Beillat M, Treur M. Efficacy, tolerability, and safety of aripiprazole once-monthly versus other long-acting injectable antipsychotic therapies in the maintenance treatment of schizophrenia: a mixed treatment comparison of double-blind randomized clinical trials. *J Mark Access Health Policy* 2015; **3**.	Overlapping RCT meta-analysis
Malham KM, Miller BJ. Long-Acting Injectable Antipsychotics and Infections in Schizophrenia. *J Clin Psychopharmacol* 2023; **43**(3): 259–62.	The reported adverse outcome was excluded from our analysis
Manoubi SA, Boussaid M, Brahim O, Ouanes S, Mahjoub Y, Zarrouk L, et al. Fatal pulmonary embolism in patients on antipsychotics: case series, systematic review and meta-analysis. *Asian J Psychiatr* 2022; **73**: 103105.	The reported adverse outcome was excluded from our analysis
Marshall M, Rathbone J. Early intervention for psychosis. *Cochrane Database Syst Rev* 2011; (6): CD004718.	Overlapping RCT meta-analysis and reported other outcomes excluded from our analysis
Maruki T, Utsumi T, Takeshima M, Fujiwara Y, Matsui M, Aoki Y, et al. Efficacy and safety of adjunctive therapy to lamotrigine, lithium, or valproate monotherapy in bipolar depression: a systematic review and meta-analysis of randomized controlled trials. *Int J Bipolar Disord* 2022; **10**(1): 24.	The reported adverse outcomes were excluded from our analysis
McQuire C, Hassiotis A, Harrison B, Pilling S. Pharmacological interventions for challenging behaviour in children with intellectual disabilities: A systematic review and meta-analysis. *BMC Psychiatry* 2015; **15**(1): 303.	Overlapping RCT meta-analysis and reported other outcomes excluded from our analysis
Meduri M, Gregoraci G, Baglivo V, Balestrieri M, Isola M, Brambilla P. A meta-analysis of efficacy and safety of aripiprazole in adult and pediatric bipolar disorder in randomized controlled trials and observational studies. *J Affect Disord* 2016; **191**: 187–208.	Overlapping RCT meta-analysis and reported other outcomes excluded from our analysis
Myles N, Myles H, Xia S, Large M, Kisely S, Galletly C, et al. Meta-analysis examining the epidemiology of clozapine-associated neutropenia. *Acta Psychiatr Scand* 2018; **138**(2): 101–9.	Unable to calculate OR or RR for reported outcomes
Nashed MG, Restivo MR, Taylor VH. Olanzapine-induced weight gain in patients with bipolar I disorder: A meta-analysis. *Primary Care Companion to the Journal of Clinical Psychiatry* 2011; **13**(6).	The reported adverse outcomes were excluded from our analysis
Nishi A, Sawada K, Uchida H, Mimura M, Takeuchi H. Antipsychotic Monotherapy for Major Depressive Disorder: A Systematic Review and Meta-Analysis. *Pharmacopsychiatry* 2023; **56**(1): 5–17.	Overlapping RCT meta-analysis and reported other outcomes excluded from our analysis
Nussbaum AM, Stroup TS. Drug information update: Paliperidone palmitate for schizophrenia. *Psychiatrist* 2013; **37**(5): 164–6.	Overlapping RCT meta-analysis and reported other outcomes excluded from our analysis
Oderda LH, Young JR, Asche CV, Pepper GA. Psychotropic-related hip fractures: meta-analysis of first-generation and second-generation antidepressant and antipsychotic drugs. *Ann Pharmacother* 2012; **46**(7/8): 917–28.	Overlapping observational study meta-analysis: not the most recently published
Orr C, Deshpande S, Sawh S, Jones PM, Vasudev K. Asenapine for the Treatment of Psychotic Disorders: A Systematic Review and Meta-analysis. *Can J Psychiatry* 2017; **62**(2): 123–37.	Overlapping RCT meta-analysis
Ostuzzi G, Bertolini F, Tedeschi F, Vita G, Brambilla P, Del Fabro L, et al. Oral and long-acting antipsychotics for relapse prevention in schizophrenia-spectrum disorders: a network meta-analysis of 92 randomized trials including 22,645 participants. *World Psychiatry* 2022; **21**(2): 295–307.	Overlapping RCT meta-analysis and reported other outcomes excluded from our analysis
Oya K, Kishi T, Iwata N. Efficacy and tolerability of aripiprazole once monthly for schizophrenia: A systematic review and meta-analysis of randomized controlled trials. *Neuropsychiatr Dis Treat* 2015; **11**: 2299–307.	Overlapping RCT meta-analysis and reported other outcomes excluded from our analysis
Pagsberg AK, Tarp S, Glintborg D, Stenstrom AD, Fink-Jensen A, Correll CU, et al. Acute Antipsychotic Treatment of Children and Adolescents With Schizophrenia-Spectrum Disorders: A Systematic Review and Network Meta-Analysis. *J Am Acad Child Adolesc Psychiatry* 2017; **56**(3): 191–202.	Overlapping RCT meta-analysis and reported other outcomes excluded from our analysis
Prajapati AR, Wilson J, Song F, Maidment I. Second-generation antipsychotic long-acting injections in bipolar disorder: Systematic review and meta-analysis. *Bipolar Disord* 2018; **20**(8): 687–96.	Overlapping RCT meta-analysis and reported other outcomes excluded from our analysis
Pringsheim T, Lam D, Ching H, Patten S. Metabolic and neurological complications of second-generation antipsychotic use in children: A systematic review and meta-analysis of randomized controlled trials. *Drug Saf* 2011; **34**(8): 651–68.	Overlapping RCT meta-analysis and reported other outcomes excluded from our analysis
Rodrigues R, Lai MC, Beswick A, Gorman DA, Anagnostou E, Szatmari P, et al. Practitioner Review: Pharmacological treatment of attention-deficit/hyperactivity disorder symptoms in children and youth with autism spectrum disorder: a systematic review and meta-analysis. *J Child Psychol Psychiatry Allied Discip* 2020; **62**(6): 680–700.	Overlapping RCT meta-analysis and reported other outcomes excluded from our analysis
Rodrigues R, Lai MC, Beswick A, Gorman DA, Anagnostou E, Szatmari P, et al. Practitioner Review: Pharmacological treatment of attention-deficit/hyperactivity disorder symptoms in children and youth with autism spectrum disorder: a systematic review and meta-analysis. *J Child Psychol Psychiatry Allied Discip* 2021; **62**(6): 680–700.	Overlapping RCT meta-analysis and reported other outcomes excluded from our analysis
Ruxton K, Woodman RJ, Mangoni AA. Drugs with anticholinergic effects and cognitive impairment, falls and all-cause mortality in older adults: A systematic review and meta-analysis. *Br J Clin Pharmacol* 2015; **80**(2): 209–20.	Overlapping observational study meta-analysis: not the most recently published
Sampson S, Hosalli P, Furtado VA, Davis JM. Risperidone (depot) for schizophrenia. *Cochrane Database Syst Rev* 2016; **4**(4): CD004161.	Overlapping RCT meta-analysis and reported other outcomes excluded from our analysis
Siafis S, Ç ıray O, Wu H, Schneider-Thoma J, Bighelli I, Krause M, et al. Pharmacological and dietary-supplement treatments for autism spectrum disorder: a systematic review and network meta-analysis. *Mol Autism* 2022; **13**(1): 10.	Overlapping RCT meta-analysis
Silva MT, Zimmermann IR, Galvao TF, Pereira MG. Olanzapine plus fluoxetine for bipolar disorder: A systematic review and meta-analysis. *J Affect Disord* 2013; **146**(3): 310–8.	Overlapping RCT meta-analysis
Singappuli P, Sonuga-Barke E, Kyriakopoulos M. Antipsychotic long-term treatment in children and young people: a systematic review and meta-analysis of efficacy and tolerability across mental health and neurodevelopmental conditions. *CNS Spectr* 2022; **27**(5): 570–87.	Overlapping RCT meta-analysis and reported other outcomes excluded from our analysis
Siskind D, Sidhu A, Cross J, Chua YT, Myles N, Cohen D, et al. Systematic review and meta-analysis of rates of clozapine-associated myocarditis and cardiomyopathy. *Aust N Z J Psychiatry* 2020; **54**(5): 467–81.	Unable to calculate OR or RR for reported outcomes
Spielmans GI, Berman MI, Linardatos E, Rosenlicht NZ, Perry A, Tsai AC. Adjunctive atypical antipsychotic treatment for major depressive disorder: a meta-analysis of depression, quality of life, and safety outcomes. *PLoS Med* 2013; **10**(3): e1001403-e.	Overlapping RCT meta-analysis and reported other outcomes excluded from our analysis
Srisurapanont M, Maneeton B, Maneeton N, Lankappa S, Gandhi R. Quetiapine for schizophrenia. *Cochrane Database Syst Rev* 2004; (2): CD000967.	Overlapping RCT meta-analysis and reported other outcomes excluded from our analysis
Stafford MR, Mayo-Wilson E, Loucas CE, James A, Hollis C, Birchwood M, et al. Efficacy and safety of pharmacological and psychological interventions for the treatment of psychosis and schizophrenia in children, adolescents and young adults: A systematic review and meta-analysis. *PLoS One* 2015; **10**(2): e0117166.	Overlapping RCT meta-analysis and reported other outcomes excluded from our analysis
Stoffers-Winterling JM, Storebø OJ, Pereira Ribeiro J, Kongerslev MT, Völlm BA, Mattivi JT, et al. Pharmacological interventions for people with borderline personality disorder. *Cochrane Database Syst Rev* 2022; **11**(11): CD012956.	Overlapping RCT meta-analysis and reported other outcomes excluded from our analysis
Stogios N, Smith E, Bowden S, Tran V, Asgariroozbehani R, McIntyre WB, et al. Metabolic adverse effects of off-label use of second-generation antipsychotics in the adult population: a systematic review and meta-analysis. *Neuropsychopharmacology* 2022; **47**(3): 664–72.	Overlapping RCT meta-analysis and reported other outcomes excluded from our analysis
Suttajit S, Srisurapanont M, Maneeton N, Maneeton B. Quetiapine for acute bipolar depression: A systematic review and meta-analysis. *Drug Des Devel Ther* 2014; **8**: 827–38.	Overlapping RCT meta-analysis and reported other outcomes excluded from our analysis
Tan ECK, Lexomboon D, Sandborgh-Englund G, Haasum Y, Johnell K. Medications That Cause Dry Mouth As an Adverse Effect in Older People: A Systematic Review and Metaanalysis. *J Am Geriatr Soc* 2018; **66**(1): 76–84.	The reported adverse outcome was excluded from our analysis
Tek C, Kucukgoncu S, Guloksuz S, Woods SW, Srihari VH, Annamalai A. Antipsychotic-induced weight gain in first-episode psychosis patients: A meta-analysis of differential effects of antipsychotic medications. *Early Interv Psychiatry* 2016; **10**(3): 193–202.	Overlapping RCT meta-analysis and reported other outcomes excluded from our analysis
Vancampfort D, Vansteelandt K, Correll CU, Mitchell AJ, De Herdt A, Sienaert P, et al. Metabolic syndrome and metabolic abnormalities in bipolar disorder: a meta-analysis of prevalence rates and moderators. *Am J Psychiatry* 2013; **170**(3): 265–74.	Overlapping observational study meta-analysis: not the most recently published
Vázquez GH, Bahji A, Undurraga J, Tondo L, Baldessarini RJ. Efficacy and Tolerability of Combination Treatments for Major Depression: Antidepressants plus Second-Generation Antipsychotics vs. Esketamine vs. Lithium. *J Psychopharmacol* 2021; **35**(8): 890–900.	Overlapping RCT meta-analysis and reported other outcomes excluded from our analysis
Vita A, De Peri L, Siracusano A, Sacchetti E. Efficacy and tolerability of asenapine for acute mania in bipolar I disorder: Meta-analyses of randomized-controlled trials. *Int Clin Psychopharmacol* 2013; **28**(5): 219–27.	Overlapping RCT meta-analysis and reported other outcomes excluded from our analysis
Wang D, Schneider-Thoma J, Siafis S, Burschinski A, Dong S, Wu H, et al. Long-Acting Injectable Second-Generation Antipsychotics vs Placebo and Their Oral Formulations in Acute Schizophrenia: A Systematic Review and Meta-Analysis of Randomized-Controlled-Trials. *Schizophr Bull* 2023: sbad089.	Overlapping RCT meta-analysis and reported other outcomes excluded from our analysis
Wang F, Wen F, Yu L, Yan J, Liu J, Li Y, et al. The efficacy and safety in attention deficit hyperactivity disorder of second-generation antipsychotics and other medications for hyperactivity in children and adolescents with autism: a meta-analysis. *Int Clin Psychopharmacol* 2021; **36**(3): 109–16.	Overlapping RCT meta-analysis and reported other outcomes excluded from our analysis
Willems AE, Tenback DE, Ingenhoven TJM, van Harten PN. Acute movement disorders associated with the use of second-generation antipsychotics in borderline personality disorder: A meta-analysis. *J Clin Psychopharmacol* 2014; **34**(4): 520–2.	Overlapping RCT meta-analysis
Yu ZH, Jiang HY, Shao L, Zhou YY, Shi HY, Ruan B. Use of antipsychotics and risk of myocardial infarction: a systematic review and meta-analysis. *Br J Clin Pharmacol* 2016; **82**(3): 624–32.	Overlapping observational study meta-analysis: not the most recently published
Zhai Y, Yin S, Zhang D. Association between Antipsychotic Drugs and Mortality in Older Persons with Alzheimer's Disease: A Systematic Review and Meta-Analysis. *J Alzheimers Dis* 2016; **52**(2): 631–9.	Overlapping observational study meta-analysis: not the most recently published
Zhang R, Dong L, Shao F, Tan X, Ying K. Antipsychotics and venous thromboembolism risk: A meta-analysis. *Pharmacopsychiatry* 2011; **44**(5): 183–8.	Overlapping observational study meta-analysis: not the most recently published
Zhao MJ, Qin B, Wang JB, Zhang YP, Zhao JT, Mao YG, et al. Efficacy and Acceptability of Cariprazine in Acute Exacerbation of Schizophrenia: Meta-Analysis of Randomized Placebo-Controlled Trials. *J Clin Psychopharmacol* 2018; **38**(1): 55–9.	Overlapping RCT meta-analysis

## Appendix 2

Search strategy for PubMed.

(antipsychotic agents/ae OR antipsychotic agents/to OR antipsychotic agents/po

OR chlorpromazine[tiab] OR fluphenazine[tiab] OR haloperidol[tiab] OR

pimozide[tiab] OR prochlorperazine[tiab] OR trifluoperazine[tiab] OR

benperidol[tiab] OR flupentixol[tiab] OR levomepromazine[tiab] OR

pericyazine[tiab] OR promazine[tiab] OR sulpiride[tiab] OR zuclopenthixol[tiab] OR

acepromazine[tiab] OR droperidol[tiab] OR loxapine[tiab] OR triflupromazine[tiab]

OR perphenazine[tiab] OR thioridazine[tiab] OR molindone[tiab] OR

thiothixene[tiab] OR aripiprazole[tiab] OR clozapine[tiab] OR olanzapine[tiab] OR

quetiapine[tiab] OR risperidone[tiab] OR amisulpride[tiab] OR paliperidone[tiab]

OR ziprasidone[tiab] OR asenapine[tiab] OR cariprazine[tiab] OR lurasidone[tiab])

AND (((((systematic review[tiab] OR systematic literature review[tiab] OR

systematic scoping review[tiab] OR systematic narrative review[tiab] OR

systematic qualitative review[tiab] OR systematic evidence review[tiab] OR

systematic quantitative review[tiab] OR systematic meta-review[tiab] OR

systematic critical review[tiab] OR systematic mixed studies review[tiab] OR

systematic mapping review[tiab] OR systematic cochrane review[tiab] OR

systematic search and review[tiab] OR systematic integrative review[tiab]) NOT

comment[pt] NOT (protocol[tiab] OR protocols[tiab])) NOT MEDLINE [subset]) OR

(Cochrane Database Syst Rev[ta] AND review[pt]) OR systematic review[pt]) OR

(meta-analysis[pt] OR meta-analysis[tiab] OR metaanaly*[tiab] OR metaanaly*[tiab])).

Search strings comprised database-specific indexing terms (e.g. MeSH terms) attached to adverse effect-related subheadings (e.g. antipsychotic agents/ae, to, po for PubMed; and neuroleptic agent/ae, to, tm, po for Embase).

Search strategy for Embase.

1. neuroleptic agent/ae, to, tm, po.

2. (haloperidol or pimozide or prochlorperazine or trifluoperazine or benperidol or zuclopenthixol or acepromazine or droperidol or loxapine or triflupromazine or perphenazine or thioridazine or molindone or thiothixene or aripiprazole or clozapine or olanzapine or quetiapine or risperidone or amisulpride or flupentixol or levomepromazine or pericyazine or promazine or sulpiride or chlorpromazine or fluphenazine or paliperidone or ziprasidone or asenapine or cariprazine or lurasidone).ti,ab.

3. 1 or 2.

4. exp review/.

5. (literature adj3 review$).ti,ab.

6. exp meta analysis/.

7. exp Systematic Review/.

8. or/4–7.

9. (medline or medlars or embase or pubmed or cinahl or amed or psychlit or psyclit or psychinfo or psycinfo or scisearch or cochrane).ti,ab.

RETRACTED ARTICLE/.

9 or 10.

12. 8 and 11.

13. (systematic$ adj2 (review$ or overview)).ti,ab.

14. (meta?anal$ or meta anal$ or meta-anal$ or metaanal$ or metanal$).ti,ab.

15. 12 or 13 or 14.

16. 3 and 15.

Search strategy for PsycINFO.

1. exp Neuroleptic Drugs/.

2. exp "Side Effects (Drug)"/.

3. (haloperidol or pimozide or prochlorperazine or trifluoperazine or benperidol or zuclopenthixol or acepromazine or droperidol or loxapine or triflupromazine or perphenazine or thioridazine or molindone or thiothixene or aripiprazole or clozapine or olanzapine or quetiapine or risperidone or amisulpride or flupentixol or levomepromazine or pericyazine or promazine or sulpiride or chlorpromazine or fluphenazine or paliperidone or ziprasidone or asenapine or cariprazine or lurasidone).ti,ab.

4. exp review/.

5. exp "systematic review"/.

6. exp meta analysis/.

7. (literature adj3 review$).ti,ab.

8. or/4–7.

9. (medline or medlars or embase or pubmed or cinahl or amed or psychlit or psyclit or psychinfo or psycinfo or scisearch or cochrane).ti,ab.

RETRACTED ARTICLE/.

11. 9 or 10.

12. 8 and 11.

13. (systematic$ adj2 (review$ or overview)).ti,ab.

14. (meta?anal$ or meta anal$ or meta-anal$ or metaanal$ or metanal$).ti,ab.

12 or 13 or 14.

16. 1 and 2.

17. 16 or 3.

18. 17 and 15.

Search strategy for Scopus.

(TITLE-ABS(antipsychotic* OR "antipsychotic agent" OR anti-psychotic* OR "anti-psychotic agent" OR neuroleptic* OR "neuroleptic agent") AND TITLE-ABS(("adverse drug reaction" OR "side effect" OR safety OR toxicity) OR ((adverse OR undesirable OR harm* or serious or toxic) W/3 (effect* or reaction* or event* or outcome*))) OR TITLE-ABS(haloperidol OR pimozide OR prochlorperazine OR trifluoperazine OR benperidol OR zuclopenthixol OR acepromazine OR droperidol OR loxapine OR triflupromazine OR perphenazine OR thioridazine OR molindone OR thiothixene OR aripiprazole OR clozapine OR olanzapine OR quetiapine OR risperidone OR amisulpride OR flupentixol OR levomepromazine OR pericyazine OR promazine OR sulpiride OR chlorpromazine OR fluphenazine OR paliperidone OR ziprasidone OR asenapine OR cariprazine OR lurasidone)) AND TITLE-ABS((systematic W/2 (review* or overview)) OR (meta?anal* or meta anal* or meta-anal* or metaanal*)).

Search strategy for CINAHL.

((MH Antipsychotic Agents+/AE/PO) OR (TI ( haloperidol or pimozide or prochlorperazine or trifluoperazine or benperidol or zuclopenthixol or acepromazine or droperidol or loxapine or triflupromazine or perphenazine or thioridazine or molindone or thiothixene or aripiprazole or clozapine or olanzapine or quetiapine or risperidone or amisulpride or flupentixol or levomepromazine or pericyazine or promazine or sulpiride or chlorpromazine or fluphenazine or paliperidone or ziprasidone or asenapine or cariprazine or lurasidone) OR AB ( haloperidol or pimozide or prochlorperazine or trifluoperazine or benperidol or zuclopenthixol or acepromazine or droperidol or loxapine or triflupromazine or perphenazine or thioridazine or molindone or thiothixene or aripiprazole or clozapine or olanzapine or quetiapine or risperidone or amisulpride or flupentixol or levomepromazine or pericyazine or promazine or sulpiride or chlorpromazine or fluphenazine or paliperidone or ziprasidone or asenapine or cariprazine or lurasidone))) AND ((TI systematic review OR TI meta analysis OR AB systematic review OR AB meta analysis) OR (PT systematic review OR PT meta analysis)).

Search strategy for Cochrane Library of Systematic Reviews.

IDSearch.

#1MeSH descriptor: [Antipsychotic Agents] explode all trees and with qualifier(s): [adverse effects - AE].

#2MeSH descriptor: [Antipsychotic Agents] explode all trees and with qualifier(s): [poisoning - PO].

#3MeSH descriptor: [Antipsychotic Agents] explode all trees and with qualifier(s): [toxicity - TO].

#4(haloperidol or pimozide or prochlorperazine or trifluoperazine or benperidol or zuclopenthixol or acepromazine or droperidol or loxapine or triflupromazine or perphenazine or thioridazine or molindone or thiothixene or aripiprazole or clozapine or olanzapine or quetiapine or risperidone or amisulpride or flupentixol or levomepromazine or pericyazine or promazine or sulpiride or chlorpromazine or fluphenazine or paliperidone or ziprasidone or asenapine or cariprazine or lurasidone):ti,ab.

#5#1 OR #2 OR #3 OR #4.

Appendix 3.

Approach to overlapping reviews.

For each adverse effect association reported in reviews of RCTs on the same antipsychotic medication and population, the meta-analysis with the highest quality was selected when multiple meta-analyses reporting effect sizes for the same outcome were identified, to avoid data duplication. When there was a partial overlap or no overlap in the antipsychotic medication and population of these meta-analyses, the largest meta-analysis with the largest number of trials was selected. In circumstances where more than one meta-analysis included the same number of trials, the meta-analysis with more participants was included. Given the more inclusive inclusion criteria of meta-analyses of observational studies, the most recent meta-analysis was selected. When one or more meta-analyses were published in the same year, the meta-analysis with the largest number of studies was selected.

## Appendix 4

Deviations from protocol.

1. Asenapine, cariprazine and lurasidone were additionally included as antipsychotics of interest.
2. Pregnant women were excluded in our protocol, however this was intended to be specific to neonatal and child outcomes after in-utero exposure to antipsychotics, and meta-analyses reporting on *maternal* outcomes in antipsychotic-exposed pregnant women were deemed eligible for inclusion in this review.
3. All meta-analyses were performed using the random-effects model considering the high heterogeneity between studies.
4. Dropout rates associated with adverse effects, as an additional outcome, were excluded given the disparate reporting of cause-specific dropouts.
5. We did not assess small-study effects as data for many adverse effects were obtained from fewer than 10 primary studies and primary study data were unavailable for some adverse effects. The insufficient reporting of primary study data in some meta-analyses also precluded our evaluation on evidence certainty using the GRADE framework.

## Appendix 5

Complementary to the quality appraisal using AMSTAR 2, statistical tests for heterogeneity, excess significance bias, and prediction intervals were conducted to further assess the consistency and robustness of the included evidence.

Heterogeneity of the effect estimates across included reviews was assessed with the I^2^ statistic. The cut-offs for low, moderate and high heterogeneity were 25%, 50% and 75% respectively. For meta-analyses which reported the heterogeneity of their primary studies with the Cochran’s Q statistic, *Q* was converted into *I*^*2*^.

To detect excess significance bias, the Ioannidis test was used to examine whether the observed number of original studies with nominally significant (*p* < 0.05) results (O) was larger than the expected number of original studies with nominally significant results (E) at α = 0.05. We computed the ratio of the summary RR or OR of a meta-analysis to the RR or OR of the largest component study in the same meta-analysis, with the former and latter signifying O and E respectively (Ceraso et al., 2022). This was computed under the assumption that the effect size provided by the largest component study in each meta-analysis was the closest to the true effect and the most precise.

When the τ^2^ statistic was available, we estimated the 95% prediction interval for each summary effect estimate to account for between-study heterogeneity, as an expression for the uncertainty in the expected effect in future studies investigating the adverse health events associated with antipsychotic use (NHS Business Services Authority, 2020).

1. Kavvoura FK, McQueen MB, Khoury MJ, Tanzi RE, Bertram L, Ioannidis JP. Evaluation of the potential excess of statistically significant findings in published genetic association studies: application to Alzheimer's disease. Am J Epidemiol. 2008;168(8):855–65
2. IntHout J, Ioannidis JP, Rovers MM, Goeman JJ. Plea for routinely presenting prediction intervals in meta-analysis. BMJ Open. 2016;6(7):e010247.
